# Age‐related changes in the ease of dynamical transitions in human brain activity

**DOI:** 10.1002/hbm.24033

**Published:** 2018-03-09

**Authors:** Takahiro Ezaki, Michiko Sakaki, Takamitsu Watanabe, Naoki Masuda

**Affiliations:** ^1^ PRESTO, JST, 4‐1‐8 Honcho Kawaguchi Saitama Japan; ^2^ National Institute of Informatics, Hitotsubashi Chiyoda‐ku Tokyo Japan; ^3^ Kawarabayashi Large Graph Project, ERARO, JST, c/o Global Research Center for Big Data Mathematics, NII Chiyoda‐ku Tokyo Japan; ^4^ School of Psychology and Clinical Language Sciences University of Reading, Earley Gate, Whiteknights Road Reading United Kingdom; ^5^ Research Institute, Kochi University of Technology Kami Kochi Japan; ^6^ Institute of Cognitive Neuroscience, University College London, 17 Queen Square London WC1N 3AZ United Kingdom; ^7^ Department of Engineering Mathematics University of Bristol Clifton Bristol United Kingdom

**Keywords:** aging, energy landscape, executive function, fMRI, Ising model, resting‐state network

## Abstract

Executive functions, a set of cognitive processes that enable flexible behavioral control, are known to decay with aging. Because such complex mental functions are considered to rely on the dynamic coordination of functionally different neural systems, the age‐related decline in executive functions should be underpinned by alteration of large‐scale neural dynamics. However, the effects of age on brain dynamics have not been firmly formulated. Here, we investigate such age‐related changes in brain dynamics by applying “energy landscape analysis” to publicly available functional magnetic resonance imaging data from healthy younger and older human adults. We quantified the ease of dynamical transitions between different major patterns of brain activity, and estimated it for the default mode network (DMN) and the cingulo‐opercular network (CON) separately. We found that the two age groups shared qualitatively the same trajectories of brain dynamics in both the DMN and CON. However, in both of networks, the ease of transitions was significantly smaller in the older than the younger group. Moreover, the ease of transitions was associated with the performance in executive function tasks in a doubly dissociated manner: for the younger adults, the ability of executive functions was mainly correlated with the ease of transitions in the CON, whereas that for the older adults was specifically associated with the ease of transitions in the DMN. These results provide direct biological evidence for age‐related changes in macroscopic brain dynamics and suggest that such neural dynamics play key roles when individuals carry out cognitively demanding tasks.

## 1 INTRODUCTION

Normal aging is associated with the decline in many mental functions which affects older adults’ quality of life (Davis, Marra, Najafzadeh, & Liu‐Ambrose, 2010). While some cognitive skills (e.g., vocabulary and other crystalized knowledge about general information) are maintained with age, a clear age‐related decline has been observed in executive functions which allow for flexible and goal‐directed cognitive control by integrating diverse information (Park et al., 2002; Salthouse, 2009). Given accumulating evidence for associations between efficient integration of cognitive information and finely‐coordinated large‐scale neural dynamics (Fries, 2005; Rolls, Loh, Deco, & Winterer, 2008; Deco, Jirsa, & McIntosh, 2011; Uhlhaas & Singer, 2012; Kopell, Gritton, Whittington, & Kramer, 2014; Wang & Krystal, 2014; Watanabe & Rees, 2017), it is reasonable to assume that such age‐related deterioration of executive functions is underpinned by age‐associated changes in neural dynamics.

Theoretically, some studies have suggested crucial roles of brain dynamics in age‐related alterations of executive functions (Nakagawa, Jirsa, Spiegler, McIntosh, & Deco, 2013; Rolls & Deco, 2015). Behavioral research also suggests that cognitive decline with aging is relevant to dynamics—in particular transitory dynamics (i.e., temporal transitions in behavioral states). For example, older adults, relative to younger adults, are more distracted by task‐irrelevant information, such as their internal thoughts and external events (Hasher, Stoltzfus, Zacks, & Rypma, 1991; Gazzaley, Cooney, Rissman, & D'Esposito, 2005). These results suggest that transitions from one state to another may happen too frequently in the older‐aged brain as well as in older adults’ behavior.

Empirically, however, whether and how declines in cognitive functions are correlated with age‐related alterations in large‐scale neural dynamics are poorly understood. Previous human neuroimaging studies have reported associations between age‐related cognitive changes and static brain architecture, such as focal white/gray matter structures (Allen, Bruss, Brown, & Damasio, 2005; Raz et al., 2005; Andrews‐Hanna et al., 2007; Persson et al., 2016) and density of neurochemical substances (Berry et al., 2016). In addition, associations between age‐related cognitive changes and dynamical features of the brain, such as functional connectivity (Andrews‐Hanna et al., 2007; Damoiseaux et al., 2008; Esposito et al., 2008; Sambataro et al., 2010; Grady et al., 2010; Onoda, Ishihara, & Yamaguchi, 2012; Tomasi & Volkow, 2012; Grady, 2012; Meier et al., 2012; Ferreira & Busatto, 2013; Geerligs, Maurits, Renken, & Lorist, 2014, 2015; Madhyastha & Grabowski, 2014), signal variability (Garrett, Kovacevic, McIntosh, & Grady, 2010, 2013), 1/*f* noise (Voytek et al., 2015), patterns of brain activity during tasks (Davis, Dennis, Daselaar, Fleck, & Cabeza, 2008; Jimura & Braver, 2010), and neural oscillations (Pons, Cantero, Atienza, & Garcia‐Ojalvo, 2010; Voytek & Knight, 2015) have been investigated. However, these studies neither revealed how different parts of the brain dynamically integrate and disintegrate to create different activity patterns nor how one's brain transits among different activity patterns.

To address this fundamental question on aging brains, here, we conceptualized “ease of transitions” in neural dynamics as the rate of transitions between different major brain activity patterns that frequently appear during rest. We tested (1) whether the ease of transitions in neural dynamics is different between older versus younger adults and (2) whether the association between the executive ability and the ease of transitions in neural dynamics differs between younger and older adults. To address these questions, we focused on the default mode network (DMN; Raichle et al., 2001) and the cingulo‐opercular network (CON; Dosenbach et al., 2007). We chose the DMN and CON for two reasons. First, recent studies suggested that the executive ability of older adults depends more strongly on the DMN than that of younger adults (Duverne, Motamedinia, & Rugg, 2009; Turner & Spreng, 2015; Maillet & Schacter, 2016). Second, the CON is implicated in executive functions in older adults (Meier et al., 2012; Amer, Anderson, Campbell, Hasher, & Grady, 2016; Schmidt, Burge, Visscher, & Ross, 2016).

**Figure 1:**
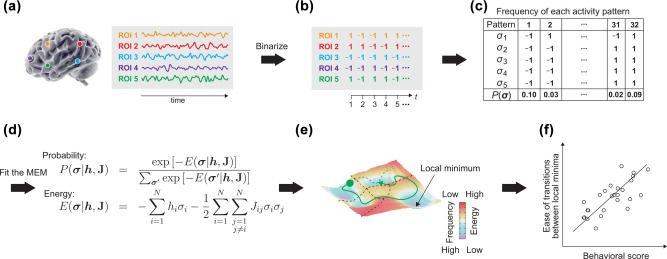
Pipeline of the energy landscape analysis. First, BOLD signals from selected ROIs (a) are binarized (b). Then, the frequency of each activity pattern is calculated (c). The distribution of the frequency of activity patterns is fitted by the MEM (d), from which an energy landscape is constructed (e). Finally, the brain dynamics quantified as the ease of transitions on the energy landscape (called “efficiency score”) is associated with a participant's behavioral score (called “executive score”), as shown in (f) [Color figure can be viewed at http://wileyonlinelibrary.com]

**Figure 2:**
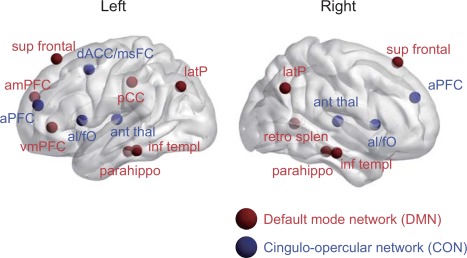
Location of the ROIs in each functional system. Retro splen: retro splenial cortex, latP: lateral parietal cortex, pCC: posterior cingulate cortex, parahippo: parahippocampal cortex, inf templ: inferior temporal cortex, sup frontal: superior frontal cortex, vmPFC: ventromedial prefrontal cortex, amPFC: anteromedial prefrontal cortex, ant thal: anterior thalamus, dACC/msFC: dorsal anterior cingulate cortex/medial superior frontal cortex, al/fO: anterior insula/frontal operculum, aPFC: anterior prefrontal cortex [Color figure can be viewed at http://wileyonlinelibrary.com]

**Figure 3:**
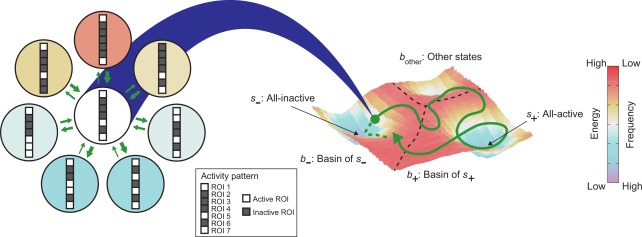
Schematic of dynamics of the activity pattern constrained on an energy landscape. The figure to the left is a blow‐up of the figure to the right around an activity pattern. Because there are seven ROIs in this example, the focal activity pattern has seven neighboring activity patterns, as shown in the left figure. A transition to a neighboring activity pattern with a lower energy (shown in blue) is more likely to occur than a transition to an activity pattern with a higher energy (shown in a warm color) [Color figure can be viewed at http://wileyonlinelibrary.com]

We characterized neural dynamics of the DMN and CON by applying a data‐driven approach called energy landscape analysis (Watanabe et al., 2014a, 2014b; Ezaki, Watanabe, Ohzeki, & Masuda, 2017; Watanabe & Rees, 2017) to resting‐state functional MRI (fMRI) signals collected from younger (18 years ≤ and ≤ 30 years) and older adults (60 years ≤ and ≤ 85 years; Table 1). Our analysis pipeline is schematically shown in Figure 1. Technically, for each brain system (Figure 2), we first inferred an energy landscape and then estimated neural dynamics as movements of a “ball” on the landscape (Figure 3). Activity patterns with a small energy value (i.e., lower positions on the landscape) appear with a high frequency. Based on the neural dynamics, we identified major brain activity patterns as those frequently visited and located at the bottom of a basin in the energy landscape, and finally quantified the ease of dynamical transitions based on the frequency with which major activity patterns were visited. Because the fronto‐parietal network (FPN) has also been implicated in aging of executive functions (Dosenbach, Fair, Cohen, Schlaggar, & Petersen, 2008), we additionally carried out the analysis on FPN but did not obtain significant results. We will discuss this point in Section 2.3.

**Table 1:** Demographic data

	Younger	Older
Age (mean ± std)	22.61 ± 2.96	70.96 ± 7.51
IQ^*^		
Performance IQ (mean ± std)	112.71 ± 11.04	111.93 ± 15.43
Verbal IQ (mean ± std)	109.61 ± 12.35	111.16 ± 9.80
Full IQ (mean ± std)	113.04 ± 11.50	111.86 ± 11.87
Female/male	14/14	14/14
Executive score^**^ (mean ± std)	0.55 ± 0.77	−0.53 ± 0.92

*IQ was evaluated by the Wechsler Abbreviated Scale of Intelligence (WASI) (Wechsler, 1999)

**Executive score was determined by the Delis‐Kaplan Executive Function System (Delis, Kaplan, & Kramer, 2001; Delis, Kramer, Kaplan, & Holdnack, 2004).

## 2 MATERIALS AND METHODS

### 2.1 Participants

We used data from 28 younger adults (19–30 years) and 28 older adults (60–85 years) from the Nathan Kline Institute's (NKI) Rockland phase I Sample (Table 1; Nooner et al., 2012): http://fcon_1000.projects.nitrc.org/indi/pro/nki.html. A similar age cut‐off has been widely used in the literature on cognitive aging (Park, Polk, Hebrank, & Jenkins, 2010, 2010b; Chadick, Zanto, & Gazzaley, 2014). The two groups were matched on race and sex, and did not significantly differ in IQ (Table 1). All of them were right handed. Data from one younger female participant were not included in the analyses of behavioral scores because she did not complete a cognitive task.

### 2.2 Behavioral data and executive score

Participants’ executive functions were assessed by the Delis‐Kaplan Executive Function System (D‐KEFS; Delis, Kaplan, & Kramer, 2001; Delis, Kramer, Kaplan, & Holdnack, 2004) which consists of multiple cognitive tests to assess executive functions. In accordance with previous literature (Latzman & Markon, 2010; Barbey et al., 2012), we constructed a composite executive score for each individual by applying a factor analysis to scores from five tests in D‐KEFS (Supporting Information Table S1): verbal fluency, sorting task, 20 questions, color word task, and design fluency. The analysis revealed three factors with the eigenvalues greater than 1 but the scree plot showed only one sharp bend in the array to eigenvalues after the first factor. Therefore, the factor score for the first factor was used as an executive score in the current study. The eigenvalue of this factor was 4.26 and it accounted for 43% of the variance with all items having positive loadings (Supporting Information Table S1). Participants’ IQ was assessed by the Wechsler Abbreviated Scale of Intelligence (WASI; Wechsler, 1999); a full scale intelligence quotient, verbal IQ, and performance IQ scores were used in the current study.

### 2.3 Definition of brain systems

We determined the regions of interest (ROIs) of the DMN and CON by employing the coordinates obtained in a previous study (Fair et al., 2009). The original systems have 12 and 7 ROIs for the DMN and CON, respectively (Figure 2). For the DMN, the pairwise maximum entropy model (MEM) did not yield a high accuracy of fit, presumably due to the insufficiency in the data length (Table 2). It should be noted that in the pairwise MEM, the accuracy of fit generally decreases with *N*_ROI_ if the amount of the observed data is fixed (Ezaki et al., 2017). Therefore, we analyzed the right‐ and left‐hemisphere DMNs (the right/left DMN with 8 ROIs, respectively) instead of the original DMN. The number of ROIs for these one‐hemisphere systems was not equal to the half of the original DMN because some ROIs (i.e., amPFC, vmPFC, pCC, and retro splen) were almost on the midline such that they were used in both the right and left DMNs.

**Table 2:** Accuracy of the fitting by the pairwise MEM. Four ROIs in the DMN in the medial part of the brain were shared by the right and left DMNs

	Right DMN	Left DMN	Whole DMN	Whole CON
*N*_ROI_	8	8	12	7
Younger	0.930	0.911	0.659	0.973
Older	0.866	0.839	0.467	0.971

### 2.4 fMRI data acquisition and preprocessing

The MRI data were recorded in a 3T scanner (MAGNETOM, TrioTim syngo MR B15, Siemens). fMRI data were obtained during rest with an echo planner imaging (EPI) sequence (TR = 2,500 ms, TE = 30 ms, flip angle = 80°, 38 slices, spatial resolution = 3 × 3 × 3 mm^3^, FOV = 216 ms, acquisition time = 10 min 55 s). Anatomical images were acquired with T1‐weighted sequence (MPRAGE; TR = 2,500 ms, TE = 3.5 ms, flip angle = 8°, spatial resolution = 1 × 1 × 1 mm^3^). During the EPI data acquisition, the participants were asked to be relaxed with their eyes open.

Data preprocessing was performed using FMRIB's Software Library (FSL; http://www.fmrib.ox.ac.uk/fsl), including skull stripping of structural images with BET, motion correction with MCFLIRT, and smoothing with full‐width half‐maximum 5 mm. Registration was performed with FLIRT; each functional image was registered to the participant's high‐resolution brain‐extracted structural image and the standard Montreal Neurological Institute (MNI) 2‐mm brain. We also applied additional preprocessing steps to the functional data to remove spurious variance. First, we regressed out six head motion parameters, global signal, cerebrospinal fluid (CSF) signal, and white matter (WM) signal with FSL FEAT. For each participant, CSF, gray matter (GM) and WM were segmented through FSL's FAST based on his/her T1. We next applied band‐pass temporal filtering (0.01–0.1 Hz). The data were then re‐smoothed by Gaussian kernels with sigma = 2.12 (the same setting as the one applied during the initial smoothing) with an FSL command line tool called SUSAN to improve the signal‐to‐noise ratio. Finally, we extracted the global signal for each volume again and subtracted this global signal from the averaged signal of all voxels within each spherical ROI (radius = 4 mm) in the DMN and CON (Watanabe et al., 2013, 2014b). In this way, we avoided overestimation of synchronization in brain activity between different ROIs.

We confirmed that the magnitude of the head motion was not significantly different between the younger and older adults (*t*_54_ = –.33, *p* = .75). We quantified the magnitude of the head motion by the average displacement of each volume relative to the previous volume, computed from the translation parameters in the *x* (left/right), *y* (anterior/posterior), and *z* (superior/inferior) directions as $\sqrt{x^{2}+y^{2}+z^{2}}$ (Van Dijk, Sabuncu, & Buckner, 2012).

### 2.5 Fitting of the pairwise MEM

We fitted the pairwise MEM to the fMRI data using a standard method as follows (Watanabe et al., 2013; Ezaki et al., 2017; Figure 1). Because the method demands a relatively large amount of data, we pooled the fMRI signals from the participants in the same age group and then fitted a pairwise MEM. Consider a system of *N*_ROI_ ROIs. For each ROI, labeled *i* (=1, …, *N*_ROI_), we denote the binarized activity at time *t* by $\sigma_{i}^{t}\;(1\leq t\leq T)$, which is equal to either +1 (active) or −1 (inactive). For each ROI in each participant, we set a threshold, above which we regarded the ROI to be active, to the average signal value for the ROI across time for the participant, resulting in approximately 50% of time points being active for the ROI for the participant. Thus, the threshold value was different across ROIs and across participants. We confirmed that the accuracy of fitting, defined in the following, is insensitive to the threshold value as long as the fraction of active time points falls within 0.25 and 0.75 (Supporting Information Figure S1). The activity pattern at time *t* is specified by an *N*_ROI_‐dimensional binary vector $\;[\sigma_{1}^{t},\;\sigma_{2}^{t},\;\ldots ,\;\sigma_{N_{ROI}}^{t}]$ (Figure 3). Note that there are $2^{N_{ROI}}$ possible activity patterns, which are enumerated as $V_{1}=$[–1, −1, …, −1], …, $V_{2^{N_{ROI}}}=$ [1, 1, …, 1].

For each ROI, we aggregated the data over time and across the participants in the same age group, and calculated the frequency that each activity pattern $V_{k}$ (*k* = 1, …, $2^{N_{ROI}}$) was realized, denoted by $P_{empirical}(V_{k})$. For the pairwise MEM, the frequency obeys the Boltzmann distribution,

(1) $$P\left(V_{k}\right)=\;e^{-E(V_{k})}/\sum_{l=1}^{2^{N_{ROI}}}e^{-E(V_{l})},$$

where $E\left(V_{k}\right)$ represents the energy for activity pattern $V_{k}$ defined by

(2) $$E\left(V_{k}\right)=\;-\sum_{i=1}^{N}h_{i}\sigma_{i}-\;(1/2)\sum_{i=1}^{N_{ROI}}\sum_{j=1}^{N_{ROI}}J_{ij}{\sigma_{i}\sigma }_{j}.$$

Here, the fitting parameters $h_{i}\;and\;\;J_{ij}$ represent the tendency for the *i*th ROI to be active when it is isolated and the strength of the interaction between the *i*th and *j*th ROIs, respectively. A small energy value corresponds to a large frequency of appearance of an activity pattern by definition.

To obtain $h_{i}\;and\;J_{ij}$ (*i*, *j* = 1, …, *N*_ROI_), we first calculated the average and pairwise correlation of the empirical data as follows:

(3) $$〈\sigma_{i}〉=\;(1/T)\;\sum_{t=1}^{T}\sigma_{i}^{t},$$

(4) $$〈\sigma_{i}\sigma_{j}〉=(1/T)\;\sum_{t=1}^{T}\sigma_{i}^{t}\sigma_{j}^{t}.$$

The average and correlation expected from the model (Equation (1)) for given $h_{i}\;and\;J_{ij}$ (*i*, *j* = 1, …, *N*) are equal to ${〈\sigma_{i}〉}_{m}=\;\sum_{k=1}^{2^{N_{ROI}}}\sigma_{i}\left(V_{k}\right)\;P(V_{k})\;\text{and}\;{〈\sigma_{i}\sigma_{j}〉}_{m}=\sum_{k=1}^{2^{N_{ROI}}}\sigma_{i}\left(V_{k}\right)\;\sigma_{j}\left(V_{k}\right)\;P(V_{k}),$ respectively. We iteratively adjusted $h_{i}\;and\;J_{ij}$ according to $h_{i}^{new}=\;h_{i}^{old}+\;\alpha \left(〈\sigma_{i}〉-{〈\sigma_{i}〉}_{m}\right)\;\text{and}\;J_{ij}^{new}=\;J_{ij}^{old}+\;\alpha \left(〈\sigma_{i}\sigma_{j}〉-{〈\sigma_{i}\sigma_{j}〉}_{m}\right)$ such that the values of ${〈\sigma_{i}〉}_{m}\;\text{and}\;{〈\sigma_{i}\sigma_{j}〉}_{m}$ gradually approach the empirical values (Equations (3) and (4)). This iterative scheme is a gradient decent method minimizing the Kullback‐Leibler divergence given by $D_{2}\;=\;\sum_{k=1}^{2^{N_{ROI}}}P_{empirical}(V_{k})\cdot \log_{2}\left(P_{empirical}(V_{k})/P_{model}(V_{k})\right)$. We set α=0.1.

### 2.6 Accuracy of fit

We used the following accuracy measure (Schneidman, Berry, Segev, & Bialek, 2006; Shlens et al., 2006; Watanabe et al., 2013, 2014a, 2014b; Ezaki et al., 2017) to assess the goodness of the fit of the pairwise MEM to the fMRI data obtained from each age group:

(5) $$r_{D}=\;\left(D_{1}-D_{2}\right)/D_{1},$$

where $D_{1}$ represents the Kullback‐Leibler divergence between the MEM and data when the MEM is restricted to have no interaction term, that is, $J_{ij}=0$ for all *i* and *j*. We obtain $r_{D}=1$ when the pairwise MEM perfectly reproduces the empirical distribution of activity patterns, whereas $r_{D}=0$ when the pairwise interaction (i.e., $J_{ij}$) does not contribute to improve the fitting.

### 2.7 Disconnectivity graph

For each age group, we calculated a disconnectivity graph (Becker & Karplus, 1997; Wales, Miller, & Walsh, 1998; Wales, 2010) from the estimated pairwise MEM in the same way as was done in our previous studies (Watanabe et al., 2014a, 2014b; Watanabe & Rees, 2017). In the network of activity patterns, where each activity pattern constitutes a node, activity patterns $V_{k}$ and $V_{k'}$ were defined to be adjacent (i.e., directly connected by an edge) if they were the same across all ROIs except for one. Therefore, each activity pattern was adjacent to *N* activity patterns. For example, if *N*_ROI_ = 3, activity pattern [1, 1, 1] is adjacent to [–1, 1, 1], [1, −1, 1], and [1, 1, −1]. Then, we identified the activity patterns whose energy values were smaller than all of their *N*_ROI_ adjacent activity patterns, that is, local minima in the energy landscape (bottom of a basin in Figure 3). Local minima are the activity patterns that are more likely to appear than all their neighboring patterns.

We obtained the disconnectivity graph from the network of activity patterns as follows. First, we identified all local minima by exhaustively examining whether each activity pattern was a local minimum. Second, we set an energy threshold value, denoted by *E*_th_, to the energy value of the activity pattern that attained the second largest energy value among the $2^{N_{ROI}}$ activity patterns. Third, we removed the nodes corresponding to the activity patterns whose energy exceeded *E*_th_. When *E*_th_, is the second largest energy, the node with the largest energy was removed. We also removed the edges incident to the removed nodes. Fourth, we checked whether each pair of local minima was connected in the reduced network. Fifth, we lowered *E*_th_ to the next largest energy value realized by an activity pattern. Then, we repeated the third to fifth steps, that is, removal of the nodes and edges, checking of connectivity between local minima, and lowering of *E*_th_, until all the local minima were isolated. In the course of the procedure, we obtained for each pair of local minima the largest *E*_th_ value at which the two local minima were disconnected. This *E*_th_ value is equal to the energy barrier that the dynamics of the brain have to overcome to reach from one local minimum to the other. Finally, we constructed a hierarchical tree whose terminal leaves represented the local minima. The vertical positions of these leaves represent their energy values. Those of the branches represent the height of the energy barrier that separates the local minima belonging to the two branches.

### 2.8 Attractive basin of a local minimum

The attractive basin of an energy local minimum for each age group was computed as follows (Stillinger & Weber, 1984; Becker & Karplus, 1997; Zhou, 2011; Watanabe et al., 2014a, 2014b; Ezaki et al. 2017; Watanabe & Rees, 2017). First, we selected a node *i* in the network of activity patterns. If the selected node was not a local minimum, we moved to the node with the smallest energy value among the nodes adjacent to the currently visited node. We repeated moving downhill in this manner until a local minimum was reached. The initial node *i* belongs to the basin of the finally reached local minimum. We ran this procedure for each initial node *i*.

### 2.9 Index of ease of dynamic transitions: Efficiency score

For each network, we denote the two synchronized activity patterns by *s*_+_ = [1, 1, …, 1] (i.e., all ROIs are active) and *s*_–_ = [–1, −1, …, −1] (i.e., all ROIs are inactive). For each individual, we defined the rate of transitions between *s*_+_ and *s*_–_ as the sum of the number of times that the activity $\left[\sigma_{1}^{t},\;\sigma_{2}^{t},\;\ldots ,\;\sigma_{N_{ROI}}^{t}\right]$ has left *s*_+_ and arrived at *s*_–_ before revisiting *s*_+_, and the number of times that $\left[\sigma_{1}^{t},\;\sigma_{2}^{t},\;\ldots ,\;\sigma_{N_{ROI}}^{t}\right]$ has left *s*_–_ and arrived at *s*_+_ before revisiting *s*_–_, divided by the number of volumes.

**Figure 4:**
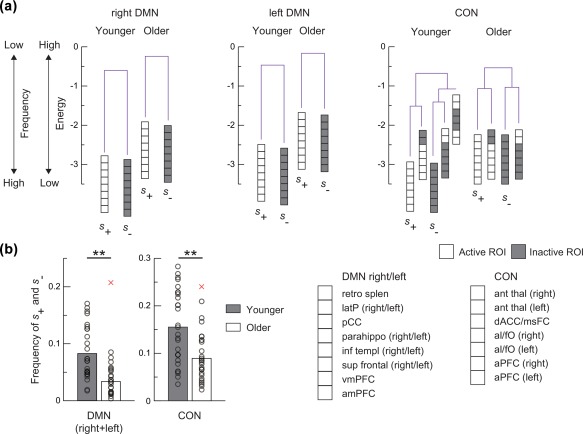
(a) Disconnectivity graph for each system and age group. (b) Frequency of *s*_+_ and *s*_–_ for each system and age group. Each symbol and bar represent the results for a participant and their average, respectively. **p_Bonferroni_ < .01, in two‐sample *t* tests (*N*_younger_ = 28 and *N*_older_ = 27). The crosses represent outliers. Note that inclusion of these outliers did not influence the statistical significance (DMN: *t*_53_ = 3.76, *p*_Bonferroni_ < 10^−3^, *d* = 1.01, CON: *t*_49_ = 3.39, *p*_Bonferroni_ < .01, *d* = 0.91 in two‐sample *t* tests) [Color figure can be viewed at http://wileyonlinelibrary.com]

We denote by *b*_+_ the attractive basin of *s*_+_ excluding *s*_+_ and by *b*_–_ the attractive basin of *s*_–_ excluding *s*_–_. Because the disconnectivity graph of the DMN had only two major activity patterns, *s*_+_ and *s*_–_ (Figure 4a), we classified all activity patterns of the DMN into the following four categories: *s*_+_, *s*_–_, *b*_+_, and *b*_–_ (Figure 6a). Because the disconnectivity graph of the CON had a few local minima in addition to *s*_+_ and *s*_–_, we classified the activity patterns of the CON into five categories: *s*_+_, *s*_–_, *b*_+_, *b*_–_, and the others (denoted by *b*_other_; Figure 6b). To define the rate of peripheral transitions for each individual, we first calculated the rate of transitions between *b*_+_ and *b*_–_ as the sum of the number of times that $\left[\sigma_{1}^{t},\;\sigma_{2}^{t},\;\ldots ,\;\sigma_{N_{ROI}}^{t}\right]$ for the individual transited from *b*_+_ to *b*_–_ and the number of times that $\left[\sigma_{1}^{t},\;\sigma_{2}^{t},\;\ldots ,\;\sigma_{N_{ROI}}^{t}\right]$ transited from *b*_–_ to *b*_+_, divided by the number of volumes. It should be noted that we used *b*_+_ and *b*_–_ that were derived from the energy landscape calculated for the age group to which the focal individual belongs, whereas we counted the transitions between *b*_+_ and *b*_–_ for the fMRI signals obtained from the individual, not the group of individuals. We refer to transitions between *b*_+_ and *b*_–_ that do not involve transitions to *s*_+_ or *s*_–_ as peripheral transitions. Precisely, the rate of peripheral transitions was defined as the rate of transitions between *b*_+_ and *b*_–_ subtracted by the rate of transitions between *s*_+_ and *s*_–_. The efficiency score for an individual was defined as the ratio of the rate of transitions between *s*_+_ and *s*_–_ to the rate of peripheral transitions. Thus, the efficiency score reflects how frequently transitions between different major brain activity patterns occur relative to peripheral transitions.

### 2.10 Numerical simulations

We carried out numerical simulations to emulate brain dynamics constrained on the estimated energy landscape. As in our previous studies (Watanabe et al., 2014a, 2014b; Watanabe & Rees, 2017), we employed the Metropolis‐Hastings algorithm (Chib & Greenberg, 1995; Zhou, 2011). First, we set the initial activity pattern to *s*_+_. Then, in each time step, a transition from the current activity pattern $V_{k}$ to one of its *N* adjacent activity patterns $V_{k'}$, selected with probability 1/*N*, was attempted. The transition to the selected pattern took place with probability $q=\min\left[1,\exp\left\{E(V_{k})-E(V_{k'})\right\}\right]$. With probability 1−q, the attempted transition was discarded. We ran the dynamics for sufficiently many time steps, that is, 10^8^, such that the initial condition did not influence the results.

### 2.11 Statistics

The age factor in our analysis of variance (ANOVA) was dichotomous (i.e., younger versus older). We made this choice because we had to pool data over participants in each age group to secure a sufficient amount of data for the present analysis.

In the analysis at the individual's level, we excluded outliers based on the Tukey's criteria of 1.5 interquartile range (Tukey, 1977). We confirmed that our main results were not affected by the choice of a method for excluding the effect of outliers (see Supporting Information).

## 3 RESULTS

### 3.1 Behavioral results

General intelligence was not significantly different between younger and older individuals (performance IQ, *F*_1,53_ = 0.05, *p* = .83; verbal IQ, *F*_1,52_ = 0.25, *p* = .25; full IQ, *F*_1,53_ = 0.14, *p* = .71; Table 1). The older adults showed a significantly lower performance than the younger adults in terms of the executive score (*F*_1,53_ = 22.2, *p* < .001, η^2^ = .22; Table 1). This result is consistent with the previous results on age‐related declines in executive functions (Park et al., 2002; Salthouse, 2009) and suggests the validity of this behavioral index.

### 3.2 Accuracy of fitting of a pairwise MEM to fMRI data

**Figure 5:**
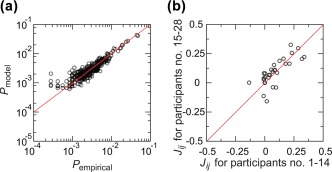
Fitting of the pairwise MEM. (a) Comparison between the probability with which each activity pattern is realized. A circle represents one of the $2^{N_{ROI}}$ activity patterns. The diagonal is shown by the solid line. *P*_empirical_ and *P*_model_ represent the probability for the empirical data and that based on the MEM, respectively. (b) Consistency between the estimated parameter values across participants. The *J_ij_* values estimated from the first half of the participants and those estimated from the second half of the participants are compared. A circle corresponds to the *J_ij_* value calculated for a pair of *i* and *j*. Both panels were based on the data obtained from the right DMN (*N*_ROI_ = 8) of the younger participants’ group [Color figure can be viewed at http://wileyonlinelibrary.com]

We analyzed resting‐state fMRI data from the younger and older individuals because such resting‐state brain activity is considered to be closely related to various cognitive functions of humans (Deco et al., 2011). After pooling the binarized fMRI data across the participants in each age group (i.e., younger/older), we fitted the pairwise MEM for each age group and each system (i.e., DMN or CON) and found that in all the cases, the model accurately fitted to the empirical fMRI signals (accuracy of fit ≥ 83%, Table 2; Figure 5a). In addition, the parameter values estimated for the pairwise MEM did not change when we estimated them for different subsets of the participants (Figure 5b), suggesting the robustness of the current method. The accuracy of fit was consistently larger for the younger than older adults (Table 2), whose implication will be discussed in the Section 4.4.

### 3.3 Identification of major activity patterns

Such accurate fitting of the pairwise MEM allows us to assign a hypothetical “energy value’’ to each of the $2^{N_{ROI}}$ activity patterns. The energy value here is not a physical quantity but a computational construct rooted in statistical physics and uniquely encodes the probability with which each activity pattern appears (Figure 3). By definition, an activity pattern with a smaller energy value occurs more frequently.

Based on the energy values assigned to all the $2^{N_{ROI}}$ activity patterns, we built and analyzed an energy landscape for each brain system (i.e., DMN or CON) for each age group (Figure 3) by creating a disconnectivity graph for each energy landscape (Figure 4). We refer to local minima of the energy as major activity patterns. The disconnectivity graph shows the height of the barrier between an arbitrary pair of major activity patterns (the smallest height that one has to ‘climb’ to move from one major activity pattern to another).

For both the right and left DMNs in both age groups, the disconnectivity graph was composed of two local minimum activity patterns, which are *s*_+_ (all ROIs are active) and *s*_–_ (all ROIs are inactive; Figure 4a). This result suggests that brain dynamics in the DMN are dominated by the synchronized activity patterns irrespectively of age.

Despite this similarity, the older group, relative to the younger group, had larger energy values (i.e., smaller frequency of appearance) at *s*_+_ and *s*_–_ and a smaller energy barrier between *s*_+_ and *s*_–_, which suggests that dynamics of transitions between *s*_+_ and *s*_–_ are different between the two groups. We corroborated this observation by carrying out an individual‐level analysis to show that the frequency of visiting *s*_+_ and *s*_–_ was significantly smaller for the older than younger adults (*t*_40_ = 5.11, *p*_Bonferroni_ < 10^−4^, Cohen's *d* = 1.36; the left panel of Figure 4b).

In contrast, the CON had more complicated disconnectivity graphs with more branches and local minima. Nevertheless, as was the same as the DMN, *s*_+_ and *s*_–_ were the most major brain activity patterns with the smallest energies (i.e., the highest frequency of appearance) in both age groups (Figure 4a), and they were visited with a higher frequency in the younger than the older group (*t*_44_ = 4.03, *p*_Bonferroni_ < 10^−3^, *d* = 1.08; the right panel of Figure 4b).

### 3.4 Quantification of ease of transitions in brain dynamics

**Figure 6:**
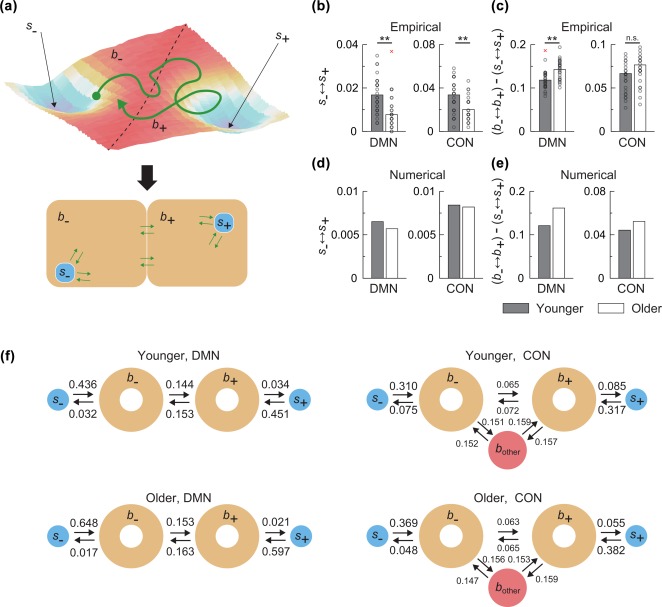
Transition rates on the energy landscape of the DMN and CON. (a) Schematic showing the procedure to categorize the $2^{N_{ROI}}$ activity patterns into four groups in the case of the DMN. The figure on the top depicts a hypothetical energy landscape, similar to Figure 3, and the dynamics of the activity pattern shown by the movement of a ball. The synchronized activity patterns, *s*_+_ and *s*_–_, are local minima. The dotted line divides the basins of attraction for *s*_+_ and *s*_–_, that is, *b*_+_ and *b*_–_. We classified the activity patterns into these four groups. Note that *s*_+_ and *s*_–_ are composed of a single synchronized activity pattern. The probability flow between activity patterns is depicted in the figure to the bottom. We aggregate the probability flow from activity patterns in *b*_–_ to those in *b*_+_, for example, to obtain the probability flow from group *b*_–_ to *b*_+_. (b)–(e) Transition rates compared between the younger and older groups. (b) Rate of transitions between *s*_+_ and *s*_–_ for the two systems and two participant groups, calculated from the empirical data. (c) Rate of peripheral transitions calculated from the empirical data. (d) Rate of transitions between *s*_+_ and *s*_–_ constructed from the numerically simulated data. (e) Rate of peripheral transitions calculated from the numerically simulated data. In (b)–(e), the bars represent the group‐averaged results. In (b) and (c), a circle represents a participant. A cross represents an outlier. (f) Conditional transition probability (i.e., transition rate divided by the probability that the group of the activity patterns in question is visited) between each possible pair of *s*_+_, *s*_–_, *b*_+_, *b*_–_, and *b*_other_ in the DMN and CON for the two age groups. See Supporting Information for the results separately obtained for the right and left DMNs. **p_Bonferroni_ < .01, in two‐sample *t* tests (*N*_younger_ *= N*_older_ = 28 before excluding outliers). Note that the inclusion of the outliers did not influence the statistical significance reported in the main text (main effects of Age: (b) *F*_1,54_ = 25.1, *p* < 10^−5^, η^2^ = .34, (c) *F*_1,54_ = 18.4, *p* < 10^−4^, η^2^ = .06 in two‐way factorial Age × System ANOVAs) [Color figure can be viewed at http://wileyonlinelibrary.com]

We hypothesized that the age‐related differences in brain dynamics, or more specifically, ease of transitions (i.e., the rate of transitions between different major activity patterns), are linked to individuals’ performance in executive functioning. To test this hypothesis, we used the inferred energy landscapes to quantify ease of transitions in the brain dynamics for each brain system and each age group. We then calculated frequencies of transitions between the four categories of activity patterns (DMN) or five categories (CON) for each individual. We did so by directly investigating time series of the empirical data for each individual (Figure 6b,c) and by performing random‐walk numerical simulations for each age group to verify the validity of the present approach (Figure 6d,e).

For the empirical fMRI data, we found that in both of the DMN and CON, the individuals in the younger group showed more frequent transitions between *s*_+_ and *s*_–_ than those in the older group did (*F*_1,53_ = 34.8, *p* < 10^−6^, η^2^ = .17 for the main effect of Age in ANOVA [Age: younger/older] × [System: DMN/CON] on the rate of transitions between *s*_+_ and *s*_–_; Figure 6b). In contrast, the individuals in the older group showed more frequent “peripheral transitions”, that is, transitions between *b*_+_ and *b*_–_ that did not involve transitions to *s*_+_ or *s*_–_ (see Section 2 for the definition; *F*_1,53_ = 25.1, *p* < 10^−5^, η^2^ = .06 for the main effect of Age in another two‐way factorial ANOVA [Age: younger/older] × [System: DMN/CON] on the peripheral transition score; Figure 6c). In older adults, once their brain activity pattern exits from *s*_+_ or *s*_–_, the activity pattern tends to fluctuate between basins *b*_+_ and *b*_–_ without easily reaching *s*_+_ or *s*_–_ (Figure 6f). The rank order of these results was reproduced by group‐level numerical simulations of the random‐walk model, which supports the validity of the present analysis method (Figure 6d,e). Altogether, these results imply that brain dynamics of younger adults are more efficient than those of older adults in the sense of ease of transitions between *s*_+_ or *s*_–_.

**Figure 7:**
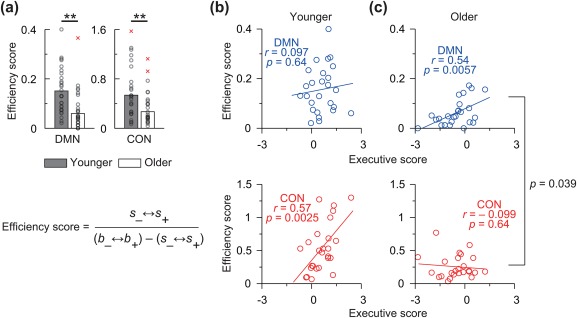
(a) Efficiency score, which measures the ease of transitions between the synchronized activity patterns, compared between the two age groups for the DMN and CON. The bars represent average values excluding the outliers shown by the crosses. A circle and cross represent a (non‐outlier) participant and an outlier, respectively. The efficiency scores from one younger adult and two older adults were identified as outliers for the CON, and the efficiency score from one additional older adult was identified as an outlier for the DMN. **p_Bonferroni_ < .01 in post‐hoc two‐sample *t*‐tests. The inclusion of the outliers marginally influenced the significance of the age‐by‐system interaction (*F*_1,54_ = 3.92, *p* = .053 in a two‐way factorial Age × System ANOVA) but did not influence the main effect of Age (*F*_1,54_ = 14.4, *p* < 10^−3^, η^2^ = .08). Panels (b) and (c) show the relationship between the efficiency score and the executive score. (b) Younger group. (c) Older group. A symbol represents a participant. The Pearson's correlation coefficient, denoted by *r*, was calculated for each age group. The linear regression is shown by the lines. A significant difference was found in the correlation coefficient (*r*) between the DMN and CON in the older group (*t* = 2.2, *p* = .039, *r* = .43, *N*_older_ = 25), whereas no significant difference was found in the younger group (*t* = 1.74, *p* = .096, *N*_younger_ = 26). See Supporting Information for the results separately obtained for the right and left DMNs [Color figure can be viewed at http://wileyonlinelibrary.com]

We confirmed the implications of these results by quantifying the efficiency of brain dynamics for each individual using an “efficiency score’’. The efficiency score was defined as the ratio of the rate of transitions between *s*_+_ and *s*_–_ to the rate of peripheral transitions. Four participants (one younger individual and three older individuals) were identified as outliers in terms of the efficiency score. After excluding these four participants, we found that the efficiency score was smaller in the older group than the younger group, in both DMN and CON (main effect of Age: *F*_1,49_ = 21.6, *p* < 10^−4^, η^2^ = .12 in a two‐way factorial Age × System ANOVA; Figure 7a). The same ANOVA also revealed a significant age‐by‐system interaction (*F*_1,49_ = 4.53, *p* < .05, η^2^ = .03). This interaction reflects the fact that the difference in the efficiency score between the DMN and CON (DMN – CON) was significantly larger in the older group than the younger group (*t*_37_ = 2.15, *p* < .05, *d* = 0.60 in a post‐hoc two‐sample *t* test), suggesting that compared to the CON, the ease of transitions in the DMN dynamics less deteriorates with aging.

### 3.5 Association between efficiency of brain dynamics and executive ability

Then, we tested whether the efficiency score predicts the executive score. We found that the executive score for the younger adults was significantly correlated with the efficiency score for the CON (*r* = .57, *p* = .0025, *p*_Bonferroni_ < .05, *df* = 24) but not with the efficiency score for the DMN (*r* = –.097, *p* = .64, *df* = 24; Figure 7b). The older adults showed the opposite pattern: the executive score was not significantly correlated with the efficiency score for the CON (*r* = –.099, *p* = .64, *df* = 23), whereas it was significantly correlated with the efficiency score for the DMN (*r* = .54, *p* = .0057, *p*_Bonferroni_ < .05, *df* = 23). These results were robust against some variation in the threshold for binarizing fMRI signals, in particular in the DMN (Supporting Information Figure S4).

In addition, the correlation coefficient between the executive score and the efficiency score was significantly larger for the DMN than the CON in the older group (*t*_22_ = 2.20, *p* = .039) but not in the younger group (*t*_23_ = −1.74, *p* = .096; Figure 7c). It should be noted that the difference between the correlation coefficients was assessed by the William's *t* test for comparing two nonindependent correlations with a variable in common (Weaver & Wuensch, 2013). Even after including the four outliers, the older adults still showed a stronger correlation between their executive score and efficiency score for the DMN than for the CON (*t*_25_ = 2.04, *p* = .05), whereas the younger adults did not (*t*_25_ = −0.26, *p* = .80).

These results suggest that the executive ability of younger adults is related to the efficiency in brain dynamics of the CON rather than that of the DMN, whereas the executive ability of older adults relies on the DMN efficiency rather than on the CON efficiency. In contrast, the frequency of visiting the synchronized activity patterns (i.e., *s*_+_ or *s*_–_), a simpler index to characterize large‐scale dynamics in the brain, did not consistently predict the executive score in the two age groups (DMN, younger: *r* = .16, *p* = .41, *df* = 25; CON, younger: *r* = .22, *p* = .26, *df* = 25; DMN, older: *r* = .38, *p* = .049, *df* = 26; CON, older: *r* = –.014, *p* = .47, *df* = 26; all correlations, *p*_Bonferroni_ > .05).

### 3.6 Association between functional connectivity and executive ability

**Figure 8:**
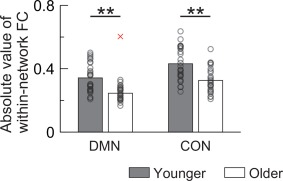
Average functional connectivity compared between the younger and older groups. The circles and the cross represent the averages of the absolute value of the functional connectivity over all ROI pairs in a brain system (i.e., DMN or CON) for (non‐outlier) individuals and the outlier, respectively. The bars represent the group averages calculated without the outlier. **p_Bonferroni_ < 10^−2^ in post‐hoc two‐sample *t* tests. The inclusion of the outlier did not influence the statistical significance of the results (main effect of Age: *F*_1,54_ = 23.6, *p* < 10^−4^, η^2^ = .54, age‐by‐system interaction: *F*_1,54_ = 0.28, *p* = .60 in a two‐way factorial ANOVA, [Age: younger/older] × [System: DMN/CON]) [Color figure can be viewed at http://wileyonlinelibrary.com]

As a control analysis, we compared the within‐system functional connectivity (FC) between the younger and older groups. The within‐system FC was estimated as an averaged Pearson correlation over all pairs of ROIs belonging to each brain system (i.e., DMN or CON). The average of FC was calculated after applying Fisher's Z transformation to raw Pearson correlation values. In both DMN and CON, the within‐network FC significantly declined with age (main effect of age group: *F*_1,53_ = 36.2, *p* < 10^−6^, η^2^ = .23 in a two‐way factorial ANOVA, [Age: younger/older] × [System: DMN/CON]; Figure 8). However, the interaction between the age and brain system was not significant (*F*_1,53_ = 0.086, *p* = .77). The FC was not significantly correlated with the executive score, either (DMN, younger: *r* = .18, *p* = .37, *df* = 25; CON, younger: *r* = .27, *p* = .17, *df* = 25; DMN, older: *r* = .33, *p* = .085, *df* = 26; CON, older: *r* = .12, *p* = .53, *df* = 26; uncorrected *p* values).

We also compared DMN‐CON connectivity between the two age groups. The FC between the DMN and the CON was estimated as the Pearson correlation averaged over all pairs of ROIs one of which belongs to the DMN and the other to the CON. The DMN‐CON FC declined with age (*t*_54_ = 2.4, *p* = .019, *d* = 0.65). This result is consistent with a previous study (Petrican, Taylor, & Grady, 2017). However, the DMN‐CON FC was not significantly correlated with the executive score (younger: *r* = .057, *p* = .78, *df* = 25; older: *r* = .13, *p* = .52, *df* = 26).

## 4 DISCUSSION

By applying the energy landscape analysis to resting‐state fMRI data, we quantified “ease of transitions” in the intrinsic human brain dynamics and found its correlate with the executive functions of younger and older adults. While major activity patterns were similar between the different age groups, transitions between them were less frequent in the older than the younger adults, which was reflected in a significantly lower efficiency score of neural dynamics for the older adults. Such a decline in the efficiency score due to aging was smaller at the DMN than the CON. In addition, we found that in the older adults, the efficiency score at the DMN, but not the CON, was correlated with their executive performance. By combining these findings, we suggest that brain dynamics in the DMN are critical to support older adults’ executive functioning and that ease of transitions in neural dynamics may be essential for sustaining their executive performances.

### 4.1 DMN, CON, and other brain systems

We found that the ease of dynamical transitions observed in the CON's neural activity was correlated with the executive score in the younger but not older group. This result is consistent with previous observations suggesting critical roles of the CON in executive control (Dosenbach et al., 2008, 2007). In contrast, the ease of transitions in the DMN was correlated with the executive score in the older group specifically. These findings support the hypothesis that, differently from younger adults, older individuals heavily rely on the DMN rather than the CON for cognitive functions (Maillet & Schacter, 2016). A major support for this hypothesis has come from reduced deactivation within the DMN during cognitively demanding tasks, such as a difficult working memory task or semantic classification task (Maillet & Schacter, 2016). Our results for the DMN are consistent with this hypothesis.

We focused on the DMN and CON based on previous studies that predicted age‐related changes in brain activity of the two brain systems (Maillet & Schacter, 2016). As a control, we analyzed an auditory network (Power et al., 2011), which should be less relevant to executive functioning and found no significant correlation between the efficiency and executive scores (see Supporting Information). These results suggest that the observed effects are specific to networks relevant to executive functioning.

Because the fronto‐parietal network (FPN) has also been implicated in the effects of aging on executive functioning (Dosenbach et al., 2008), we also carried out the same set of analysis for the right‐hemispheric and left‐hemispheric FPNs but did not find the correlation between the executive score and the efficiency score (see Supporting Information). Such dissociated results between the CON and FPN may be due to their differential roles for higher‐order cognitive functions. In fact, previous studies suggest that the CON and FPN have different time scales in top‐down control (Dosenbach et al., 2008), are involved in different stages of working memory control (Wallis, Stokes, Cousijn, Woolrich, & Nobre, 2015), and play dissociable roles in alertness (Sadaghiani & D'Esposito, 2015). Therefore, given that the current executive functioning score represents various cognitive components, future studies would have to compare neural dynamics of the CON and FPN using brain activity data during more specific psychological tasks.

Our energy landscape analysis method can treat only a relatively small number of ROIs. For this reason, we have not analyzed various other brain systems whose documented numbers of ROIs is large (Fair et al., 2009; Power et al., 2011). Furthermore, we have not analyzed inter‐connected brain systems because the number of ROIs in such an inter‐connected system is large. Yet aging is suggested to induce changes in structural and functional connectivity at a whole‐brain level, either positive or negative, which may be related to modulation in information integration/channeling across modalities in aging (Andrews‐Hanna et al., 2007; Allen et al., 2011; Meier et al., 2012; Chan, Park, Savalia, Petersen, & Wig, 2014; Geerligs et al., 2014; Spreng, Stevens, Viviano, & Schacter, 2016). Another consistently observed pattern is hemispheric asymmetry reduction in older adults (Cabeza, 2002). A large amount of data or a new technique is required to advance the applicability of the energy landscape analysis to combinations of brain systems as well as to a single brain system with a large number of ROIs. This is a main limitation of the current approach.

### 4.2 Energy landscape analysis and other methods for understanding brain dynamics

Signal variability analysis is a different data‐driven approach to characterize brain dynamics. Prior research showed that fMRI signals were less variable over time for older than younger adults (Garrett et al., 2010, 2013). By referring to the phenomenon called the stochastic resonance, they speculated that a large amount of noise in fMRI signals observed for younger adults may be beneficial in efficient switching between energy local minima corresponding to particular cognitive states. Although the present research did not aim to test the stochastic resonance, our results are consistent with their theory in the point that dynamical transitions between the specified activity patterns, such as *s*_+_ and *s*_–_, are easier for younger than older adults. It should be noted that we explicitly constructed energy landscapes from fMRI data, whereas energy landscapes are conceptual objects in the previously conducted signal variability analysis.

In a separate line of research, a computational study employed simulations of biophysical models and suggested that the depth of the attractive basin in an energy landscape decreases by cognitive symptoms of schizophrenia, deteriorating short‐term memory and attention (Loh, Rolls, & Deco, 2007). Our results are consistent with theirs, whereas participants’ characteristics, methodologies, and relevant spatial scales are quite different between the two studies. Other computational approaches to aging used attractor dynamics and employed biophysical modelling as well (Nakagawa et al., 2013; Rolls & Deco, 2015). In contrast, in the current study, we revealed that the ease of transitions between the major activity patterns was different between the two age groups by analyzing large‐scale brain dynamics in a data‐driven manner without biophysical modeling.

We found that in both DMN and CON the within‐network functional connectivity significantly declined with age, consistent with previous findings for the DMN (Andrews‐Hanna et al., 2007; Damoiseaux et al., 2008; Esposito et al., 2008; Grady et al., 2010; Sambataro et al., 2010; Tomasi & Volkow, 2012; Onoda et al., 2012; Ferreira & Busatto, 2013; Geerligs et al., 2014, 2015; Madhyastha & Grabowski, 2014) and the CON (Meier et al., 2012; Geerligs, Renken, Saliasi, Maurits, & Lorist, 2015). These connectivity results are consistent with our result that younger adults have shown larger synchronization than older adults in both DMN and CON (Figure 4b). However, the functional connectivity in either DMN or CON was not correlated with the executive score. In addition, the functional connectivity in the DMN increased with age in some studies or did not change with age in other studies (Biswal et al., 2010; Koch et al., 2010; Park et al., 2010a; Allen et al., 2011; Jones et al., 2011; Meier et al., 2012; Campbell, Grigg, Saverino, Churchill, and Grady, 2013; Ferreira and Busatto, 2013; Persson, Pudas, Nilsson, and Nyberg, 2014; Ward et al., 2015; Turner and Spreng, 2015). Given these mixed results on age‐related alterations in functional connectivity, the energy landscape analysis may provide an alternative promising approach toward understanding cognitive aging in the brain. We explicitly showed that as individuals age, transitions between the synchronized activity patterns are reduced. Changes in functional connectivity alone do not tell how the ease of transitions is affected.

However, the energy landscape analysis is not the only method for revealing association between brain dynamics and cognitive aging. For example, parameter values estimated for a dynamic causal model were associated with cognitive performance, and the association was stronger for older than younger adults in a couple of brain systems including the DMN (Tsvetanov et al., 2016). This result is consistent with ours. Other methods that aim to minimize confounding effects of non‐neuronal signals contained in fMRI signals may also yield similar results.

Dynamic functional connectivity is another method to track neural dynamics, in particular in fMRI data. As the name suggests, it analyzes time‐varying correlation between pairs of ROIs using sliding windows or other methods (Hindriks et al., 2016; Choe et al., 2017). In contrast, energy landscape analysis quantifies changes of a collection of fMRI signals at different ROIs over time. At each time point, the brain state in the energy landscape analysis is given by a vector summarizing whether each ROI is active or inactive, rather than connectivity between ROIs. Investigating differences between the results produced by these two methods warrants future work.

### 4.3 Age‐related changes in the brain

While we focused on younger and older adults in the paper, we carried out the same analysis using middle‐aged individuals. The results were roughly in the middle between those for the younger group and those for the older group (Supporting Information Figure S5). In accordance, the strength of the correlation between the efficiency and executive scores was not different between the DMN and the CON.

Age‐related differences exist not only in neural activity. For example, aging is typically accompanied with the changes in the gray matter volume (e.g., Allen et al., 2005; Fjell et al., 2013). However, we confirmed that our results were not confounded by the gray matter volume (Supporting Information). In addition, older adults tend to show reduced heart rate variability, which in turn can lead to larger variances and noise in the functional connectivity of the resting‐state BOLD signal (Tsvetanov et al., 2015). BOLD signals can also include vascular/respiration signals that may differ between younger and older adults (Power, Plitt, Laumann, & Martin, 2017). We were not able to assess the contribution of these factors to the present results due to the lack of the physiological data.

While the current sample showed age‐related declines in executive functions as observed in the previous studies (Park et al., 2002; Salthouse, 2009), the IQ scores were not significantly different between younger and older adults in the present study unlike some previous studies (Kaufman, Reynolds, & McLean, 1989). In a related vein, we did not find age‐related changes in the head movement, while previous studies often reported that older adults would move more (Mowinckel, Espeseth, & Westlye, 2012). These results raise a question about the generalizability of the current findings. Future research should address whether similar patterns are obtained for other independent and larger samples.

### 4.4 Methodological issues

Our analysis method implicitly assumes that the brain dynamics are completely described by the shape of the energy landscape except for stochasticity. In particular, the history of brain dynamics is assumed not to influence the next activity pattern, whereas the current activity pattern can. Such a memoryless process is called a Markov process. With a discrete time step of TR = 2,500 ms, we validated the Markovian assumption by simulating Markovian random‐walk processes whose transition probabilities were determined by the energy landscape. We found that numerically obtained transition rates (Figure 6d,e) were close to those obtained directly from the empirical data (Figure 6b,c) in both of the DMN and CON. The hidden Markov model is another approach that quantifies memoryless stochastic transitions between states and has been recently applied to neuroimaging data (Baker et al. 2014; Vidaurre et al. 2016). Comparison between such an approach and the present one warrants future work.

We did not look into the shape of disconnectivity graphs, which was different between the DMN and CON (Figure 4a). The biological reason and functional relevance of this difference are unclear. Disconnectivity graphs of the DMN and CON during tasks may provide information regarding functional relevance of different attractive basins, as in our previous study using a bistable visual perception task (Watanabe, et al. 2014b).

The maximum entropy model fitted better to the data obtained from the younger than older adults (Table 2). This was also the case for the FPN and the auditory network (Supporting Information). There are two possible reasons underlying this result. First, the younger group yielded a higher frequency of visit to the two synchronized activity patterns, which were by far the most frequently visited activity patterns. By definition, the value of the accuracy of fit would be large when the maximum entropy model accurately estimates the frequency of visit to frequent activity patterns, even if the model is somewhat inaccurate on other activity patterns. A second possible reason is that we had to pool data across individuals in the same group and heterogeneity across the individuals may be larger for the older than the younger group. In the group‐based analysis, the dependency of the accuracy of fit on the age may act as a covariate of no interest influencing the statistical results, which is a limitation of the present study. A larger amount of data to secure a high accuracy value for both groups or development of methods requiring less data will probably mitigate the problem.

The main results here were based on a set of ROIs defined in a previous study (Fair et al., 2009), whereas the supplementary results regarding the auditory network used a different and more detailed ROI set (Power et al., 2011). We did not use the finer atlas for DMN and CON (Power et al., 2011) because our method is limited to a relatively small number of ROIs (i.e., around 10 ROIs) given the current amount of data. The DMN and CON in the finer atlas are composed of 59 and 14 ROIs, respectively. Even if we focus on a single brain hemisphere, the number of ROIs in the finer atlas is still too large for our analysis. Furthermore, we should note that whichever brain atlas we use, the ROI coordinates are calculated based on neuroimaging data obtained from young adults. Therefore, the ROI coordinates used in the present study may underrepresent the brain of older adults (Geerligs, Tsvetanov, & Henson, 2017). To address this concern, we conducted a seed‐based functional connectivity analysis. We used a 4‐mm sphere ROI in the right anterior insula (*x* = 36, *y* = 16, *z* = 5) as a seed for the CON (Sadaghiani & D'Esposito, 2015) and found that this seed region showed significant connectivity with most of the coordinates used to define the CON in the present study not only in younger adults but also in older adults (see Supporting Information Figure S8 and Table S2). We also used a 4‐mm sphere ROI in the posterior cingulate (*x* = −2, *y* = −29, *z* = 39) as a seed for the DMN. Once again, we found spatial maps that were similar between the two age groups and included most of the coordinates for the DMN for both age groups. The results were qualitatively the same for the FPN, where the seed region was a 4‐mm sphere ROI in the right dorsolateral prefrontal cortex (*x* = 43, *y* = 21, *z* = 38). These results suggest that, although the ROIs used in the current study were defined based on a younger sample in a previous research (Fair et al. 2009), these ROIs are still valid for both younger and older groups in our sample.

We removed the global signal before submitting the fMRI data to our analysis pipeline. However, justification of global signal regression remains controversial. Global signal regression should be used with care when one compares groups with different characteristics of fMRI signal fluctuations (Murphy & Fox, 2017), which may apply to the present study in which we compared younger and older groups. We opted not to try different preprocessing methods because there is no gold standard and it is even difficult to select an alternative preprocessing method (Murphy & Fox, 2017).

Our results indicate that resting‐state data bring us useful information on cognitive aging. However, integrating resting‐state data with data during more specific cognitive tasks will further our understanding of neural mechanisms underlying age‐related cognitive decline (Campbell & Schacter, 2017; Geerligs & Tsvetanov, 2017; Grady, 2017).

## COMPETING FINANCIAL INTERESTS

The authors declare no competing financial interests.

## Supporting information

Additional Supporting Information may be found online in the supporting information tab for this article.

Supporting Information
