# Supplementary material for: Age‐related changes in the ease of dynamical transitions in human brain activity
Source: Hum Brain Mapp. 2018 Mar 9;39(6):2673–88. doi: 10.1002/hbm.24033 (PMC6619404; doi:10.1002/hbm.24033)
Supplement: Supplementary file 1 — Supporting Information [file HBM-39-2673-s001.pdf]

Supporting Information

Results for the fronto-parietal network (FPN)

We applied the network and energy landscape analysis to the FPN (Fair et al., 2009). The accuracy of fitting the pairwise MEM was equal to (0.71, 0.653), (0.968, 0.934), and (0.949, 0.942) for the whole FPN (N_{ROI} = 11), the right hemispheric FPN (N_{ROI} = 6), and the left hemispheric FPN (N_{ROI} = 6), respectively, where the first and second values in the parentheses are the accuracy for the younger and older groups, respectively. Therefore, similar to the DMN, the model was not accurate enough to describe the whole FPN, and we confined ourselves to the right and left FPNs in the remainder of this section.

The disconnectivity graphs for the right and left FPNs showed similar patterns to those for the DMN, except for the right FPN of the younger group (Fig. S6A). The disconnectivity graph for the right FPN of the younger group had more branches and local minimums than those of the DMN and CON. However, in all the FPNs, s_+ and s_- were the local minimums with almost the largest frequency of being visited. Therefore, we continued to use the categorization of the activity patterns into the four groups (s_+, s_-, b_+, and b_-) or five groups (s_+, s_-, b_+, b_-, and b_{other}) depending on whether there were only two local minimums (which were the synchronized activity patterns) or more than two local minimums. Figure S2 shows the rate of transitions between s_+ and s_- and that of peripheral transitions. The difference between the age groups was less significant than that for the DMN and CON reported in the main text. In addition, the efficiency score for the FPN of the individual participants was not significantly correlated with the executive score (Fig. S3). Thus, the ease of dynamical transitions in the FPN was not related to aging.

The functional connectivity within the FPN declined with the age (t_{52} = 3.54, $p < 10^{-3}$, and d = 0.95 in a two-sample t-test; Fig S7A). This result is consistent with previous results (Allen et al., 2011; Campbell et al., 2012; Meier et al., 2012; Madhyastha and Grabowski, 2014; Geerligs et al., 2015). However, the functional connectivity within the FPN was not significantly correlated with the executive score (younger: r = –.04, p = 0.84; older: r = .22, p = .26).

Results for the auditory network

As a negative control, we conducted the same analysis on the auditory network (Aud) whose coordinates of the ROIs were defined in a previous study (Power et al., 2011). We selected this brain system because its number of ROIs (i.e., N_{ROI} = 13) is similar to that of the DMN (i.e., N_{ROI} = 12) and the Aud is considered to be less relevant to executive functions than the other systems investigated in the present study. As we did for the DMN and FPN, we separately analyzed the right (N_{ROI} = 6) and left (N_{ROI} = 7) hemispheres of the Aud. The accuracy of fitting of the pairwise MEM was equal to (0.981, 0.958) and (0.972, 0.962) for the right and left hemispheric Aud, respectively, where the first and second values in the parentheses are the accuracy for the younger and older groups, respectively.

The disconnectivity graphs for the right and left Aud were similar to those for the DMN and FPN (Fig. S6B). For the right Aud of the older group, an activity pattern adjacent to s_+ and an activity pattern adjacent to s_- (denoted by s'_+ and s'_-, respectively) were the local minima. Because their deviation from s_+ or s_- was small, we computed the efficiency score based on the basins of and transitions between s'_+ and s'_-. Figure S2 shows the rate of transitions between s_+ and s_- (s'_+ and s'_- in the case of the right Aud of the older group) and that of peripheral transitions. The two age groups were not significantly different in the peripheral transition rate, whereas they were different in the rate of transitions between s_+ and s_-. In addition, the efficiency score for the Aud of the individual participants was not significantly correlated with the executive score (Figs. S3E and S3F). Therefore, we conclude that the ease of dynamical transitions in the Aud is not correlated with executive functions.

The functional connectivity within the Aud declined with the age (t_{53} = 3.10, p < .01, and d = 0.84 in a two-sample t-test; Fig S7B). However, the functional connectivity within the Aud was not significantly correlated with the executive score (younger: r = –.086, p = .67; older: r = .27, p = .17).

Effect of the gray matter volume on the efficiency score

To examine the effects of brain's structural difference between the younger and older adults, structural data were analyzed with FSL-VBM (Douaud et al., 2007, http://fsl.fmrib.ox.ac.uk/fsl/fslwiki/FSLVBM), an optimised VBM protocol (Good et al., 2001). First, brain-extracted structural images were gray matter-segmented and registered to the MNI 152 standard space using non-linear registration. The resulting images were averaged and flipped along the x-axis to create a left-right symmetric, study-specific gray matter template. Second, all native gray matter images were non-linearly registered to this study-specific template and "modulated" to correct for local expansion (or contraction) due to the non-linear component of the spatial transformation. Finally, the average gray matter volume (GMV) at each ROI were averaged over the ROIs in each system (i.e., DMN and CON) for each individual. We then regressed out the effect of the GMV of each participant on the efficiency score.

The correlation values between the efficiency score after the removal of the effect of the GMV and the executive score were similar to those reported in the main text (DMN, younger: r

= .23, p = .27; CON, younger: r = .49, p < .012; DMN, older: r = .60, p < .01; CON, older: r = –.18, p = .41; uncorrected p values). Therefore, we conclude that our main results are not confounded by the age-related strucutural differences in the brain.

Additional results with robust linear regression

To confirm that our main results were not influenced by the choice of a method of outlier exclusion, we tested another method, robust linear regression (Yohai, 1987; Koller and Stahel, 2011), and examined the correlation between the executive score and the efficiency score. We used the *lmrob* function in the robustbase package in R (Basic Robust Statistics R package version 0.92-7. URL: http://CRAN.R-project.org/package=robustbase). For each system (i.e., DMN and CON) and age group (younger and older), we evaluated the significance of the correlation between the efficiency score and the executive score (corresponding to Figs.7B and 7C in the main text). The results were consistent with those reported in the main text (DMN, younger: r = .20, p < .01; CON, younger: r = .58, p < .01; DMN, older: r = .41, p = .043; CON, older: r = .062, p = .77; uncorrected p values).

Results for seed-based analysis

In the current study, we defined brain systems based on previous findings from a younger sample (Fair et al., 2009). To justify these ROIs for our sample, we performed a seed-based functional connectivity analysis. A seed region was a 4 s-mm sphere in the posterior cingulate [x = –2, y = –29, z = 39 in the MNI template] for the DMN, a 4-mm sphere in the right anterior insula [x = 36, y = 16, z = 5 in the MNI template] for the CON (Sadaghiani and D'Esposito 2015), and a 4-mm sphere in the right dorsolateral prefrontal cortex [x = 43, y = 21, z = 38 in the MNI template] for the FPN. From each of these seed regions, the mean time series was calculated as the average of the fMRI signal over all voxels within the sphere using a command line tool called fslmeants from FSL. Multiple regression analyses were then performed for each participant using FSL FEAT. For each seed region, we estimated a linear regression model that included the seed-region time series and several nuisance variables: six motion parameters, the global signal, the signal from the white matter, and the signal from CSF. This analysis produced participant-level maps identifying the brain regions that had correlations with the seed region. For each age group, we then carried out group-level analyses using FSL's FEAT (FLAME 1) to identify regions that have positive correlations with the seed region across participants. We employed cluster-based corrections for multiple comparisons with Gaussian random field theory (Z = 2.3; cluster significance: p = .05-corrected). Voxels shown in Table S2 represent coordinates with the highest z-statistical values in each significant cluster; the coordinates reported by FSL were converted into Talairach coordinates by an MNI-to-Talairach transformation algorithm (Lancaster et al., 2007) that were used to provide labels of the nearest gray matter using the Talairach Daemon (Lancaster et al., 2000).

The functional connectivity maps for the younger and older groups are shown in Figure S8. For each of the DMN, CON, and FPN, the maps overlap to a large extent between the two age groups, and both contain the ROIs used in the main text (green dots in the figure).

REFERENCES

Allen EA et al. (2011) A baseline for the multivariate comparison of resting-state networks. *Front Syst Neurosci* 5:2.

Campbell KL, Grady CL, Ng C, Hasher L (2012) Age differences in the frontoparietal cognitive control network: Implications for distractibility. *Neuropsychologia* 50:2212–2223.

Douaud G, Smith S, Jenkinson M, Behrens T, Johansen-Berg H, Vickers J, James S, Voets N, Watkins K, Matthews PM, James A (2007) Anatomically related grey and white matter abnormalities in adolescent-onset schizophrenia. *Brain* 130:2375–2386.

Fair DA, Cohen AL, Power JD, Dosenbach NUF, Church JA, Miezin FM, Schlaggar BL, Petersen SE (2009) Functional brain networks develop from a "local to distributed" organization. *PLoS Comput Biol* 5:e1000381.

Geerligs L, Renken RJ, Saliasi E, Maurits NM, Lorist MM (2015) A brain-wide study of age-related changes in functional connectivity. *Cereb Cortex* 25:1987–1999.

Good CD, Johnsrude IS, Ashburner J, Henson RN, Friston KJ, Frackowiak RS (2001) A voxel-based morphometric study of ageing in 465 normal adult human brains. *Neuroimage* 14:21–36.

Koller M, Stahel WA (2011) Sharpening Wald-type inference in robust regression for small samples. Comput Stat Data Anal 55(8):2504–2515.

Lancaster JL et al. (2000) Automated Talairach Atlas labels for functional brain mapping. *Hum Brain Mapp* 10(3):120–131.

Lancaster JL et al. (2007) Bias between MNI and Talairach coordinates analyzed using the ICBN-152 brain template. Hum Brain Mapp 28(11):1194–1205.

Madhyastha TM, Grabowski TJ (2014) Age-related differences in the dynamic architecture of intrinsic networks. *Brain Connect* 4:231–241.

Meier TB, Desphande AS, Vergun S, Nair VA, Song J, Biswal BB, Meyerand ME, Birn RM, Prabhakaran V (2012) Support vector machine classification and characterization of age-related reorganization of functional brain networks. *Neuroimage* 60:601–613.

Power JD, Cohen AL, Nelson SM, Wig GS, Barnes KA, Church JA, Vogel AC, Laumann TO, Miezin FM, Schlaggar BL, Petersen SE (2011) Functional network organization of the human brain. *Neuron* 72:665–678.

Sadaghiani S, D'Esposito M (2015) Functional characterization of the cinglo-opercular network in the maintenance of tonic alertness. *Cereb Cortex* 25:2763–2773.

Yohai VJ (1987) High breakdown-point and high efficiency estimates for regression. *Ann Stat* 15: 642–665.

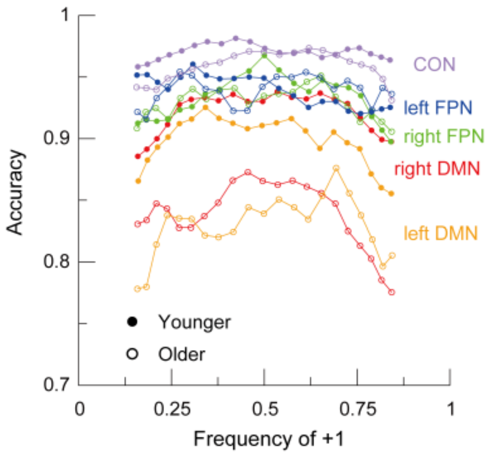

FIGURE S1 Accuracy of the fitting of the pairwise MEM for various threshold values. For each ROI and individual, we set the threshold value equal to $\mathrm{average} \pm i \times 0.1 \times \mathrm{std.}$, where *i* = 1, 2, ..., 10. The accuracy is plotted against the frequency of the +1 averaged over all ROIs.

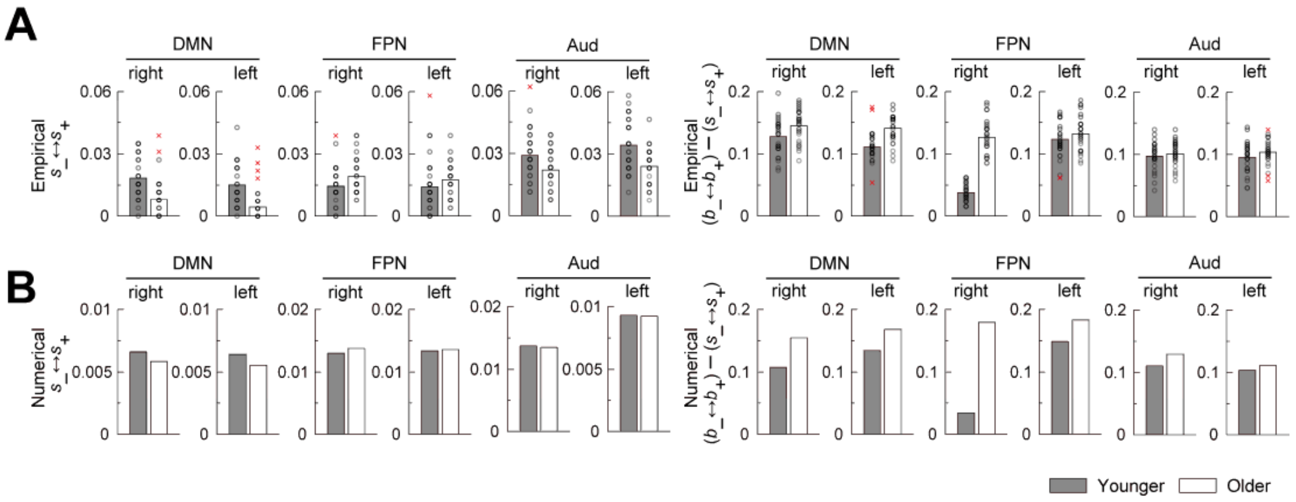

FIGURE S2 Rates of transitions between activity patterns for each hemisphere of the DMN, FPN, and Aud compared between the two age groups. (A) Empirical data. (B) Numerically simulated data. The bars represent the group-averaged results without outliers. In (A), a circle and cross represent a (non-outlier) participant and an outlier, respectively.

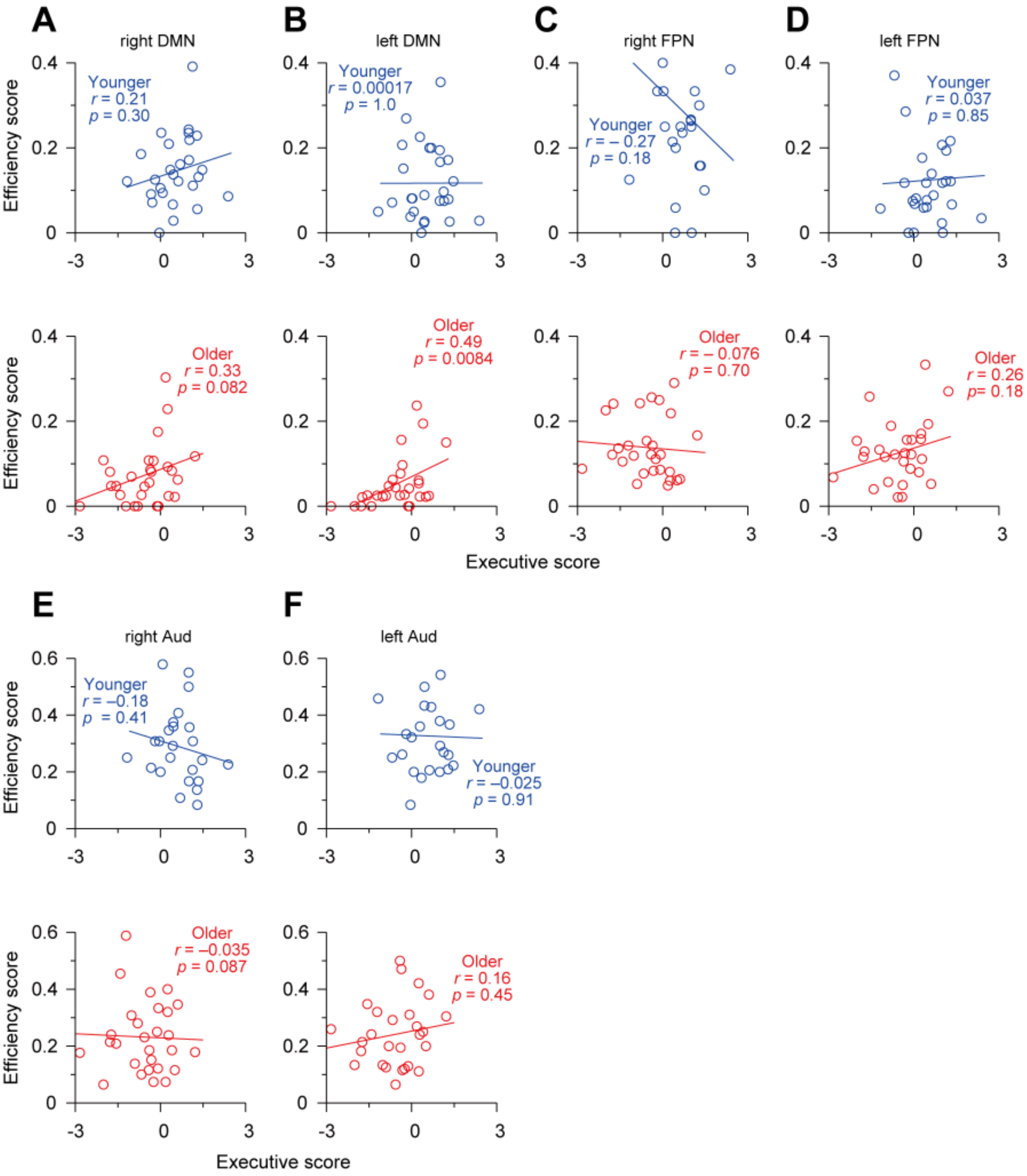

FIGURE S3 Relationship between the executive score and efficiency score for each hemisphere of the DMN, FPN, and Aud. (A) Right DMN. (B) Left DMN. (C) Right FPN. (D) Left FPN. (E) Right Aud. (F) Left Aud.

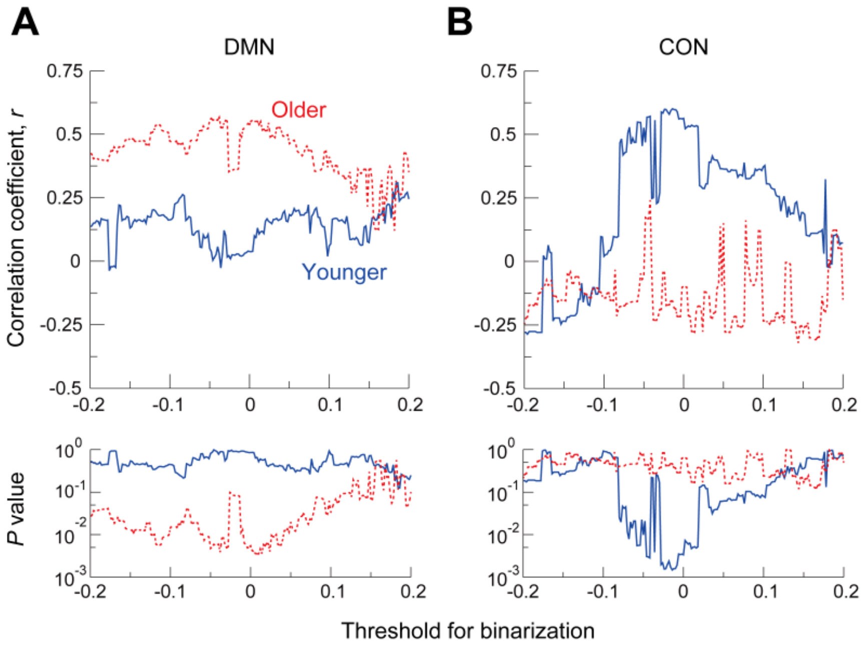

FIGURE S4 Relationship between the efficiency score and the executive score when the threshold for binarization is varied. (A) DMN. (B) CON. The *P* value is that for the significance test for the correlation coefficient.

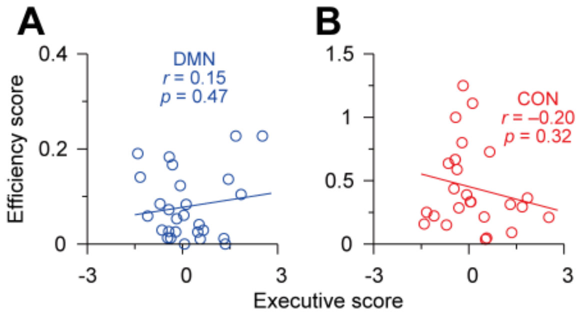

FIGURE S5 Relationship between the executive score and the efficiency score for middle-aged individuals. (A) DMN. (B) CON.

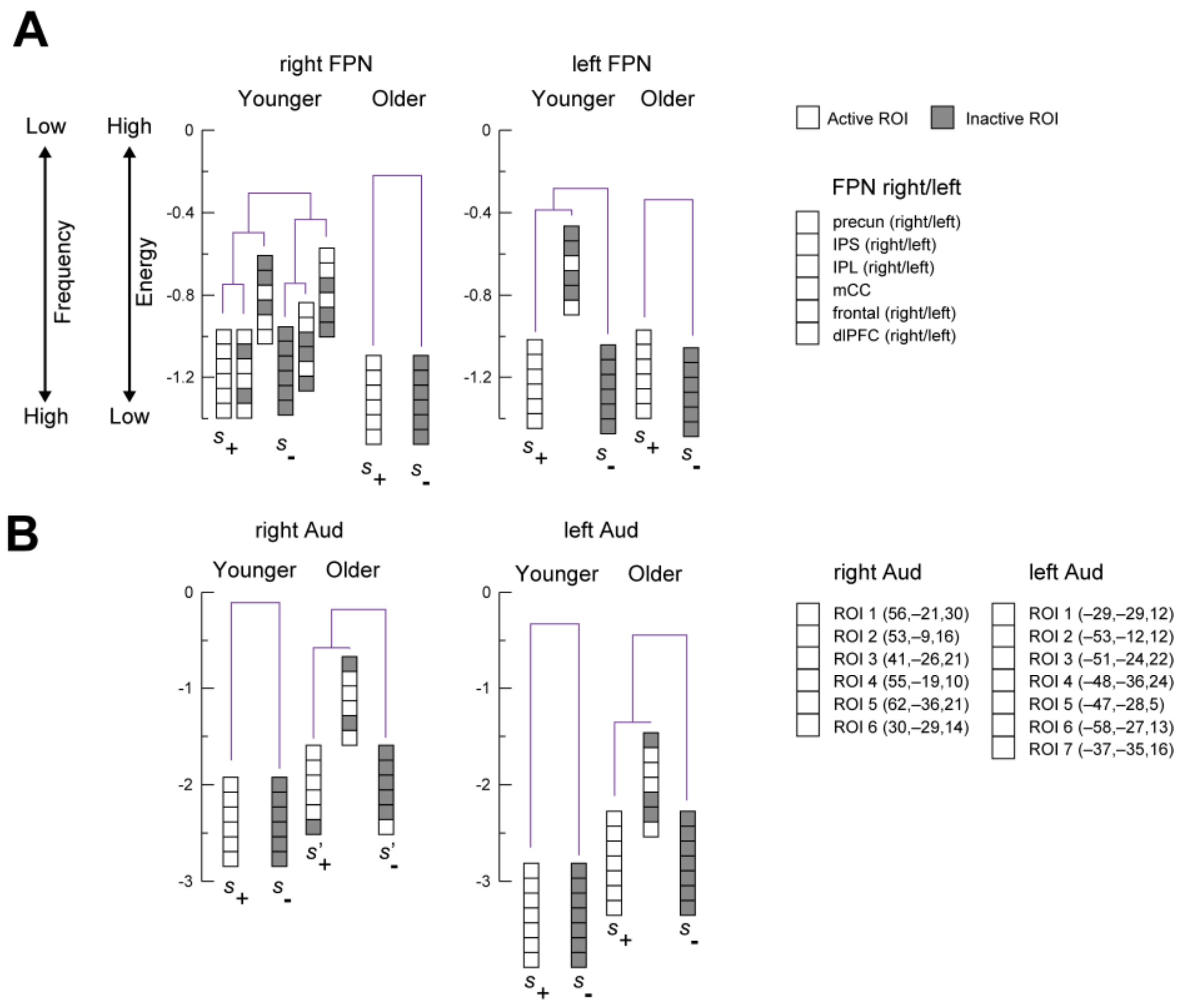

FIGURE S6 Disconnectivity graph for each hemisphere of the (A) FPN and (B) Aud for each age group. Precun: precuneus, IPS: intra-parietal sulcus, IPL: inferior parietal lobule, mCC: mid cingulate cortex, frontal: lateral frontal cortex, dlPFC: dorsolateral prefrontal cortex. For the Aud, the MNI coordinate of each ROI is shown.

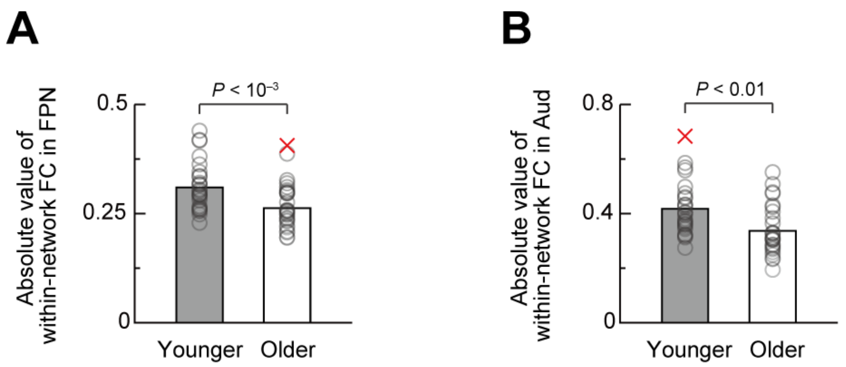

Figure S7 Average functional connectivity in the (A) FPN and (B) Aud compared between the younger and older groups. The absolute value of the functional connectivity averaged over the pairs of ROIs in each system (i.e., FPN and Aud) is shown. The bars represent the group averages without the outliers, which are shown by the crosses. The inclusion of the outlier did not influence the statistical significance of the results [(A) FPN: $t_{54} = 2.99$, $p < .01$, $d = 0.80$. (B) Aud: $t_{54} = 3.29$, $p < .01$, $d = 0.88$].

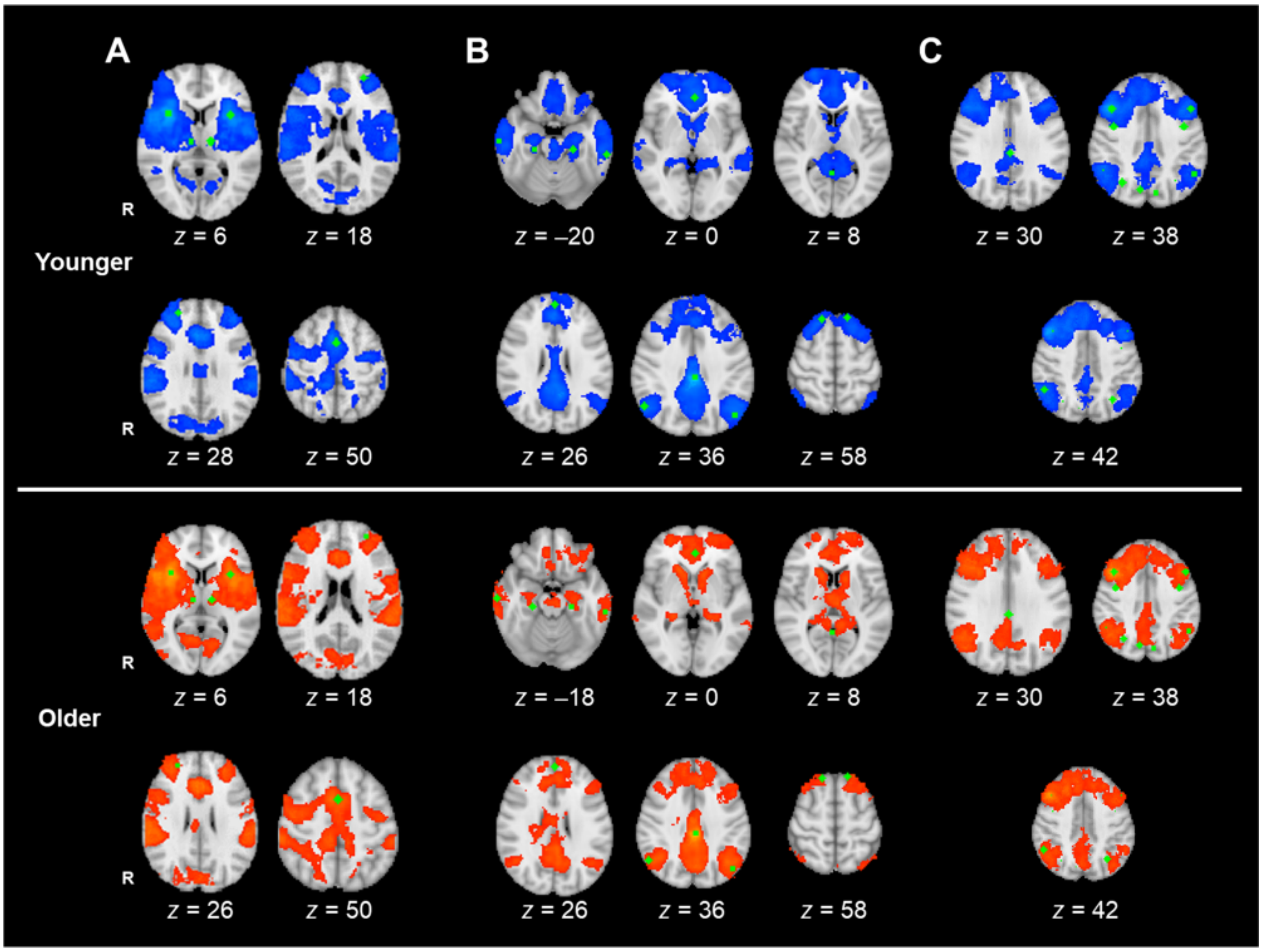

Figure S8 Results of the seed-based functional connectivity analysis for (A) CON, (B) DMN, and (c) FPN. Spatial maps that represent brain regions that were positively correlated with the seed region were shown in blue for the younger adults and red for the older adults. Green dots represent the ROIs used in the current study.

Table S1. Each subtest in D-KEFS and its factor loading score on the executive score.

		Factor loading
Verbal fluency: category fluency	Participants are asked to generate exemplars of a target category.	0.62
Verbal fluency: category switch	Participants are asked to generate exemplars of two categories in alternation.	0.59
Sort: free sort	Participants sort items into two groups and explain the categorization rules.	0.55
20 question: total questions	Participants are given illustrations of common objects and told to ask the fewest number of questions to identify the target object.	–0.40
Color-word: color naming	Participants see color patches and are asked to say the color of the ink.	–0.79
Color-word: word naming	Participants see color words printed in black and are asked to read the word.	–0.67
Color-word: inhibition	Participants see color words printed in incongruent colors and are asked to say the color of the ink.	–0.78
Design fluency: empty dots	Participants are asked to connect only empty dots to make unique designs.	0.68
Design fluency: filled dots	Participants connect dots using four straight lines to make as many unique designs as possible.	0.62
Design fluency: switching	Participants are asked to draw different designs while switching filled and unfilled dots.	0.72

Table S2. Regions showing significant results in the seed-based functional connectivity analysis for the DMN, CON, and FPN. H denotes the hemisphere.

	Number of voxels	Area	H	*x*	*y*	*z*	Z
DMN, younger	31007	Cingulate Gyrus	L	-2	-30	38	15.8
		Posterior Cingulate	L	-6	-52	26	8.32
		Posterior Cingulate	L	-4	-54	18	7.45
		Posterior Cingulate	L	-2	-42	24	7.33
		Cingulate Gyrus	L	-2	-12	34	7.22
		Cingulate Gyrus	L	-4	-46	30	7.2
	3351	Middle Temporal Gyrus	L	-64	-26	-14	6.22
		Inferior Temporal Gyrus	L	-56	-26	-18	5.97
		Middle Temporal Gyrus	L	-58	-26	-12	5.91
		Middle Temporal Gyrus	L	-58	-6	-18	5.88
		Middle Temporal Gyrus	L	-54	4	-24	5.73
		Middle Temporal Gyrus	L	-60	-34	-12	5.68
	2745	Precuneus	L	-40	-66	44	7.75
		Inferior Parietal Lobule	L	-44	-62	48	7.35
		Precuneus	L	-34	-78	48	4.81
		Superior Occipital Gyrus	L	-44	-80	38	4.33
	2438	Middle Temporal Gyrus	R	56	-22	-18	6.12
		Middle Temporal Gyrus	R	62	-22	-20	5.86
		Middle Temporal Gyrus	R	62	-26	-16	5.75
		Middle Temporal Gyrus	R	64	-18	-18	5.75
		Middle Temporal Gyrus	R	64	-14	-16	5.72
		Middle Temporal Gyrus	R	56	-2	-22	5.62
	1973	Inferior Parietal Lobule	R	50	-60	44	7.07
		Inferior Parietal Lobule	R	48	-56	48	7
		Angular Gyrus	R	48	-62	40	6.87
		Inferior Parietal Lobule	R	46	-60	44	6.84
		Inferior Parietal Lobule	R	46	-64	48	6.83
		Angular Gyrus	R	50	-62	36	6.71

	Number of voxels	Area	H	*x*	*y*	*z*	Z
DMN, younger	717	Cerebellum	L	-38	-68	-36	5.98
(continued)		Cerebellum	L	-44	-68	-36	5.59
		Cerebellum	L	-42	-62	-42	5.08
		Cerebellum	L	-38	-70	-42	5.02
		Cerebellum	L	-38	-64	-32	5.01
		Cerebellum	L	-46	-62	-36	4.77
	687	Cerebellum	R	4	-54	-48	6.28
		Cerebellum	L	-12	-52	-36	4.64
		Cerebellum	R	2	-54	-28	4.24
		Cerebellum	L	-6	-50	-36	4.14
		Cerebellum	L	-4	-56	-34	3.71
		Cerebellum	L	-4	-56	-16	3.35
	655	Cerebellum	R	38	-70	-42	5.07
		Cerebellum	R	46	-62	-38	4.89
		Cerebellum	R	44	-72	-36	4.82
		Cerebellum	R	38	-60	-40	4.78
		Cerebellum	R	34	-64	-32	4
		Cerebellum	R	44	-52	-40	4
	478	Inferior Frontal Gyrus	L	-32	34	-16	5
		Inferior Frontal Gyrus	L	-28	20	-22	4
		Claustrum	L	-28	12	-10	4
		Middle Frontal Gyrus	L	-26	30	-24	3.26
		Inferior Frontal Gyrus	L	-36	18	-18	2.77
		Claustrum	L	-30	6	-10	2.49
DMN, older	25549	Cingulate Gyrus	L	-2	-30	38	15.8
		Cingulate Gyrus	R	2	-14	36	6.59
		Precuneus	L	-4	-66	42	6.48
		Precuneus	L	-2	-68	34	6.36
		Precuneus	L	-6	-68	32	6.34
		Caudate	R	18	20	-2	6.12
	2745	Precuneus	L	-40	-62	38	6.75
		Angular Gyrus	L	-46	-60	40	6.74
		Precuneus	L	-38	-70	44	6.5
		Precuneus	L	-38	-68	40	6.45
		Angular Gyrus	L	-34	-58	40	6.04
		Inferior Parietal Lobule	L	-44	-48	40	5.2

	Number of voxels	Area	H	*x*	*y*	*z*	Z
DMN, older	1831	Inferior Parietal Lobule	R	42	-58	44	7.15
(continued)		Angular Gyrus	R	46	-60	40	6.99
		Inferior Parietal Lobule	R	48	-60	44	6.95
		Superior Parietal Lobule	R	40	-58	52	6.05
		Inferior Parietal Lobule	R	44	-56	52	5.93
		Middle Temporal Gyrus	R	54	-62	28	4.71
	1408	Middle Temporal Gyrus	L	-58	-44	-14	5.06
		Middle Temporal Gyrus	L	-56	-40	-14	4.97
		Middle Temporal Gyrus	L	-64	-30	-14	4.9
		Middle Temporal Gyrus	L	-66	-20	-12	4.82
		Middle Temporal Gyrus	L	-56	-42	-10	4.79
		Middle Temporal Gyrus	L	-56	-46	-8	4.7
	1391	Middle Temporal Gyrus	R	68	-32	-16	4.63
		Middle Temporal Gyrus	R	66	-40	-10	4.52
		Middle Temporal Gyrus	R	68	-36	-10	4.26
		Middle Temporal Gyrus	R	68	-40	-4	4.2
		Superior Temporal Gyrus	R	60	14	-18	3.93
		Middle Temporal Gyrus	R	58	-16	-22	3.85
	1089	Cerebellum	R	48	-64	-38	5.16
		Cerebellum	R	38	-68	-46	4.8
		Cerebellum	R	34	-60	-30	4.65
		Cerebellum	R	38	-60	-40	4.63
		Cerebellum	R	36	-68	-38	4.39
		Cerebellum	R	16	-82	-22	4.26
	513	Cerebellum	L	-42	-62	-42	4.9
		Cerebellum	L	-44	-68	-38	4.41
		Cerebellum	L	-38	-66	-38	4.25
		Cerebellum	L	-32	-64	-32	2.85
		Cerebellum	L	-38	-70	-28	2.8
		Cerebellum	L	-34	-72	-30	2.75

	Number of voxels	Area	H	*x*	*y*	*z*	Z
CON, younger	34311	Insula	R	36	16	4	15.2
		Insula	R	42	8	-2	10.2
		Insula	R	50	14	-4	9.37
		Claustrum	R	42	2	2	9.13
		Precentral Gyrus	R	48	10	2	8.97
		Cingulate Gyrus	R	4	24	30	8.67
	10942	Insula	L	-38	12	0	10.3
		Insula	L	-36	10	8	9.24
		Insula	L	-32	16	6	9.24
		Insula	L	-46	2	-2	8.4
		Precentral Gyrus	L	-52	-6	8	7.52
		Claustrum	L	-40	-6	-6	7.47
	1393	Middle Frontal Gyrus	L	-32	44	24	6.41
		Middle Frontal Gyrus	L	-30	48	30	6.33
		Middle Frontal Gyrus	L	-38	40	28	6.31
		Middle Frontal Gyrus	L	-38	44	32	6.24
		Middle Frontal Gyrus	L	-34	50	30	5.65
		Middle Frontal Gyrus	L	-38	38	34	5.59
CON, older	47416	Insula	R	36	16	4	15.2
		Insula	R	50	10	0	9.39
		Insula	L	-38	10	0	9.2
		Insula	L	-48	8	-4	8.88
		Insula	R	50	14	-4	8.84
		Precentral Gyrus	R	50	10	6	8.72
	448	Precentral Gyrus	L	-44	-10	50	4.16
		Precentral Gyrus	L	-40	-12	50	4.15
		Precentral Gyrus	L	-34	-8	48	3.97
		Precentral Gyrus	L	-38	-14	40	3.65
		Postcentral Gyrus	L	-52	-12	54	2.68
FPN, younger	18169	Precentral Gyrus	R	44	20	38	14.7
		Superior Frontal Gyrus	R	24	24	48	7.32
		Precentral Gyrus	L	-40	22	36	7.21
		Superior Frontal Gyrus	R	24	32	50	7
		Middle Frontal Gyrus	R	24	26	54	6.97
		Precentral Gyrus	L	-38	18	36	6.88

	Number of voxels	Area	H	*x*	*y*	*z*	Z
FPN, younger	3094	Supramarginal Gyrus	R	50	-46	38	6.45
(continued)		Angular Gyrus	R	48	-58	36	5.99
		Superior Temporal Gyrus	R	48	-54	36	5.95
		Superior Parietal Lobule	R	48	-56	54	5.68
		Inferior Parietal Lobule	R	46	-66	46	5.63
		Angular Gyrus	R	50	-64	40	5.49
	2362	Cerebellum	L	-12	-76	-26	5.51
		Cerebellum	L	-30	-70	-26	5.46
		Cerebellum	L	-14	-84	-30	4.86
		Cerebellum	L	-44	-68	-36	4.82
		Cerebellum	L	-34	-72	-42	4.72
		Cerebellum	L	-30	-70	-36	4.67
	2017	Middle Temporal Gyrus	R	64	-30	-6	5.06
		Middle Temporal Gyrus	R	60	-22	-16	4.91
		Middle Temporal Gyrus	R	64	-34	-2	4.73
		Middle Temporal Gyrus	R	60	-22	-22	4.61
		Middle Temporal Gyrus	R	60	-18	-22	4.6
		Middle Temporal Gyrus	R	60	-18	-18	4.54
	1761	Cingulate Gyrus	R	8	-38	36	6.21
		Cingulate Gyrus	R	2	-40	38	5.53
		Precuneus	R	14	-46	38	4.82
		Cingulate Gyrus	L	-8	-38	36	4.51
		Precuneus	R	16	-42	32	4.48
		Cingulate Gyrus	R	2	-26	40	4.48
	1723	Inferior Parietal Lobule	L	-48	-54	52	5.94
		Angular Gyrus	L	-44	-56	40	5.3
		Inferior Parietal Lobule	L	-46	-50	38	4.79
		Inferior Parietal Lobule	L	-46	-62	48	4.73
		Inferior Parietal Lobule	L	-50	-50	42	4.67
		Inferior Parietal Lobule	L	-44	-54	44	4.63
	707	Middle Frontal Gyrus	L	-36	58	-4	4.65
		Inferior Frontal Gyrus	L	-44	52	-6	4.33
		Middle Frontal Gyrus	L	-44	50	-12	4.16
		Inferior Frontal Gyrus	L	-48	46	-14	3.93
		Middle Frontal Gyrus	L	-40	58	-2	3.84
		Middle Frontal Gyrus	L	-28	54	-4	3.58

	Number of voxels	Area	H	*x*	*y*	*z*	Z
FPN, younger	436	Caudate	R	4	4	10	4.89
(continued)		Thalamus	L	-4	-6	16	4.4
		Thalamus	R	4	-6	16	4.24
		Caudate	R	12	6	18	4.03
		Caudate	L	-12	4	16	3.92
		Caudate	R	14	12	12	3.63
	351	Middle Temporal Gyrus	L	-66	-26	-6	4.72
		Middle Temporal Gyrus	L	-62	-22	-6	4.31
		Middle Temporal Gyrus	L	-64	-30	-10	3.94
		Middle Temporal Gyrus	L	-56	-34	-10	3.85
		Middle Temporal Gyrus	L	-60	-26	-10	3.84
		Middle Temporal Gyrus	L	-58	-34	-4	3.36
FPN, older	13084	Precentral Gyrus	R	44	20	38	14.8
		Superior Frontal Gyrus	R	26	28	46	6.89
		Middle Frontal Gyrus	R	48	28	40	6.37
		Cingulate Gyrus	R	20	22	38	6.22
		Superior Frontal Gyrus	R	24	26	40	6.18
	3775	Superior Temporal Gyrus	R	50	-52	34	6.05
		Angular Gyrus	R	48	-60	36	5.84
		Superior Temporal Gyrus	R	50	-56	34	5.77
		Angular Gyrus	R	36	-56	36	5.49
		Middle Temporal Gyrus	R	46	-66	30	5.36
		Superior Temporal Gyrus	R	58	-56	20	5.24
	2448	Precuneus	R	8	-64	32	5.04
		Precuneus	R	4	-68	42	4.42
		Precuneus	L	-12	-64	30	4.37
		Cingulate Gyrus	R	2	-44	32	4.15
		Cingulate Gyrus	R	2	-40	28	4.08
		Precuneus	L	-2	-68	42	4.07
	1603	Precuneus	L	-38	-70	42	4.6
		Inferior Parietal Lobule	L	-54	-50	46	4.41
		Supramarginal Gyrus	L	-56	-52	38	4.09
		Precuneus	L	-34	-62	38	4.04
		Middle Temporal Gyrus	L	-50	-64	24	3.95
		Inferior Parietal Lobule	L	-54	-46	46	3.94

	Number of voxels	Area	H	*x*	*y*	*z*	Z
FPN, older	1076	Caudate Body	R	16	18	6	5.77
(continued)		Caudate Body	R	16	14	10	5.29
		Thalamus	R	10	-8	8	4.71
		Caudate Body	R	10	4	12	4.13
		Caudate Body	R	8	0	16	4.09
		Caudate Body	R	14	0	14	4.07
	692	Caudate Body	L	-12	14	8	5.85
		Caudate Body	L	-16	8	16	4.73
		Caudate Body	L	-10	2	14	4.67
		Thalamus	L	-10	-10	14	4.04
		Putamen	L	-20	8	0	3.96
		Putamen	L	-18	2	4	3.36
	628	Cerebellum	L	-12	-74	-26	4.82
		Cerebellum	L	-18	-82	-30	4.32
		Cerebellum	L	-14	-78	-32	4.05
		Cerebellum	L	-34	-66	-44	3.96
		Cerebellum	L	-30	-70	-36	3.79
		Cerebellum	L	-28	-62	-36	3.66
	305	Middle Temporal Gyrus	L	-66	-36	-6	4.78
		Middle Temporal Gyrus	L	-64	-48	4	4.41
		Middle Temporal Gyrus	L	-62	-48	0	3.7
		Middle Temporal Gyrus	L	-66	-38	-2	3.42
		Middle Temporal Gyrus	L	-62	-48	-6	2.92
		Middle Temporal Gyrus	L	-58	-50	-6	2.73
